# Oxygen Evolution on Mechanically Strained TiO_2_/NiTi: Implications of Compositional Heterogeneity at (Photo)electrocatalytic Interfaces

**DOI:** 10.1021/acselectrochem.5c00228

**Published:** 2025-07-31

**Authors:** O. Quinn Carvalho, Nikita S. Dutta, Debjit Ghoshal, Steven P. Harvey, Ross A. Kerner, Shuya Li, Zebulon G. Schichtl, Patrick Walker, Logan M. Wilder, Gustavo Z. Girotto, Maximilian Jaugstetter, Slavomir Nemsak, Ethan J. Crumlin, Elisa M. Miller, Ann L. Greenaway

**Affiliations:** † Materials, Chemistry, & Computational Science Directorate, 53405 National Renewable Energy Laboratory, Golden, Colorado 80401, United States; ‡ The Advanced Light Source, 1666 Lawrence Berkeley National Laboratory, Berkeley, California 94705, United States; § Graduate Program in Physics, Federal University of Rio Grande do Sul, Porto Alegre, RS 90010-150, Brazil; ∥ Chemical Sciences Division, 1666 Lawrence Berkeley National Laboratory, Berkeley, California 94705, United States; ⊥ Department of Physics and Astronomy, University of California, Davis, Davis, California 95616, United States

**Keywords:** heterogeneous electrocatalysis, photoelectrochemistry, electrochemistry, surface characterization, oxygen evolution reaction

## Abstract

The adsorption and activation energetics underpinning small molecule conversion on heterogeneous (photo)­electrocatalysts are intrinsically tied to catalyst surface properties. Absent compositional characterization techniques with sufficient interface sensitivity, however, (photo)­electrochemical performance can be misinterpreted in the context of bulk or near-surface material properties. Here we provide a fundamental investigation of the convoluting role of near-surface compositional heterogeneity in the interpretation of (photo)­electrochemical alkaline oxygen evolution reaction (OER) activity, highlighting challenges in correlating composition measured by surface- and near-surface-sensitive probes. TiO_2_ thin films grown by air-annealing the superelastic alloy Nitinol (TiO_2_/NiTi) crack under tensile mechanical strain, increasing the number of electrochemically active Ni sites (Ni site density) that are probed via voltammetric features corresponding to Ni^3+^/Ni^2+^ redox events. (Photo)­electrochemical OER kinetics trend with Ni site density, with overpotentials and Tafel slopes decreasing for Ni site densities < 10^13^ Ni/cm^2^_geo_ and asymptotically approaching the performance of the base NiTi substrate for Ni site densities > 10^13^ Ni/cm^2^_geo_. Photoelectrochemical fill factors follow similar Ni site density dependent trends. When probing unstrained TiO_2_/NiTi, Ni site densities are two orders of magnitude higher when comparing near-surface-sensitive techniques (e.g., X-ray photoelectron spectroscopy (XPS), time of flight secondary ion mass spectrometry (TOF-SIMS), and scanning transmission electron microscopy-energy dispersive X-ray spectroscopy (STEM-EDS)) to surface-sensitive electrochemical measurements. This result highlights the challenge of correlating kinetic performance with intrinsic surface properties of electrochemical interfaces in the presence of near-surface compositional heterogeneity. Further, it reinforces the importance of fundamental investigations of surfaces with well-controlled composition and structure and the need for physically grounded and self-consistent interpretation of multiple near-surface characterization techniques.

## Graphical abstract

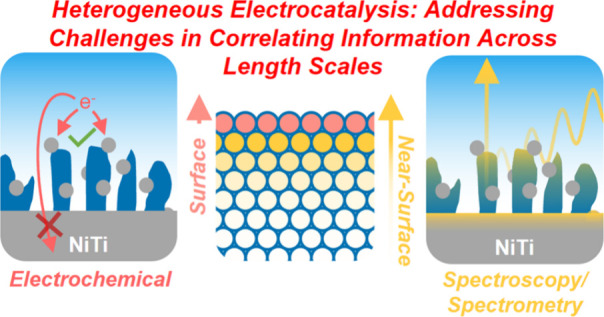

## Introduction

Lower cost grid electricity^1^ has introduced opportunities to electrify industrial energy and commodity-chemical production.^2^ One approach toward electrification is aqueous (photo)­electrocatalysis,^3–5^ where electrons with tunable chemical potential—controlled by external bias or internal photovoltage—combine with ubiquitous protons and other small molecules at heterogeneous solid-liquid interfaces to drive electrochemical reactions. Catalytic rates depend on the ability of solid (photo)­electrode surfaces to stabilize the adsorption of reactive intermediates,^6,7^ where interactions between adsorbate and surface electronic structure dictate adsorption energy.^8–10^ The composition of the surface-most atoms of the solid (photo)­electrode then serves as a key design parameter for the rational design of catalyst architectures.

The extent to which even small compositional variance influences (photo)­electrochemical reaction kinetics is well documented.^11,12^ For example, photoelectrochemical performance of rutile TiO_2_ varies with the introduction of transition metal catalysts^13,14^ and dopants.^15^ Intrinsic measures of electrochemical kinetics vary with composition on rutile oxide structures,^16^ where oxygen evolution reaction (OER) Tafel slopes measured on alloys of IrO_2_ and RuO_2_ trend systematically between behavior characteristic of the two pure components.^17^ OER kinetics on Ni-based electrodes are enhanced by the trace-level Fe contamination found in alkaline salts or from dissolution of glassware,^18^ where alloyed Ni–Fe oxyhydroxide catalysts have become a familiar benchmark for non-precious metal anodes.^19–21^ Even idealized single-crystalline surfaces are influenced by the extreme chemical potentials that occur at reactive heterogeneous interfaces, where Ni exsolution,^22,23^ Sr leaching,^24,25^ and restructuring^12,26,27^ of model perovskite oxide surfaces all capture the complex nature of electrochemically active surfaces.

Understanding electrocatalytic active sites is hindered by the lack of compositional information about the interface under reactive electrochemical conditions. Measurements of current density at heterogeneous electrochemical interfaces are inherently surface sensitive, representing the ensemble average rate of Faradaic and capacitive processes occurring between the surface-most atoms of an assumed compositionally homogeneous solid electrode and a separate electrolyte phase. Throughout our discussion, we adopt nomenclature distinguishing between the surface as the outer-most layer of atoms on a solid surface, the near-surface as the surface-most several nm, and the bulk as material lying deeper than the near-surface, even within the thin films described here.^28^ Electrochemical techniques that characterize active site density (sites/cm^2^) *in situ* are often challenged by the need for either an element-specific redox event to occur (e.g. metal redox), or for an adsorbate to selectively ad/desorb from the targeted site (e.g. hydrogen underpotential deposition, oxygen or carbon monoxide stripping, and Cu or Hg underpotential deposition).^29,30^

Spectroscopic and spectrometric tools are commonly employed *ex situ* as more generalized probes of near-surface composition, where the surface and near-surface regions are assumed to be compositionally similar. However, accurate comparisons of surface and near-surface compositional information relies on the assumption of well-behaved material systems with smooth interfaces and homogeneous compositional distribution throughout the information depth of the probe.^31^ In contrast, the presence of rough interfaces and, particularly, compositional heterogeneity within the near-surface region introduce challenges when attempting to identify whether constituent atoms exist at the electrochemically active surface or are buried within the electrochemically inactive near-surface layer.

Compositional characterization techniques, broadly delineated as being sensitive to either the near-surface or bulk, are challenged by either interface sensitivity or detection limits/modes. Bulk sensitive techniques, such as scanning electron microscopy-energy dispersive spectroscopy (SEM-EDS) and Raman and Fourier transform-infrared spectroscopy (FTIR), have characteristically long information depths (tens of nm to several μm^32–35^ ) and often lack sufficiently sensitive detection limits (>10^13^ atoms/cm^2^, e.g., SEM-EDS and Raman) or modes (e.g. Raman and FTIR). While surface and detection sensitivity may be improved by combining variations in sample-probe geometry (e.g. infrared reflection absorption spectroscopy (IRRAS), surface enhanced Raman spectroscopy (SERS))^36,37^ and through the introduction of specifically adsorbing probe molecules (e.g. carbon monoxide, pyridine),^38,39^ such improvements come at the cost of increased measurement complexity and interpretation.

In contrast, near-surface sensitive probes such as X-ray photoelectron spectroscopy (XPS) and time-of-flight secondary ion mass spectrometry (TOF-SIMS) provide compositional information from the near-surface region,^31,40–42^ with detection limits capable of capturing trace level contaminants (10^7^ atoms/cm^2^ for TOF-SIMS,^43^ ∼10^12^ atoms/cm^2^ for XPS^44^). Scanning transmission electron microscopy (STEM) may also provide both interface-sensitive microscopic and spectroscopic information when paired with EDS or electron energy loss spectroscopy (EELS) and performed on a sufficiently thin cross section of sample.^45^ Despite their near-surface sensitivity and satisfactory detection limits, the vacuum-based nature of XPS, TOF-SIMS, and STEM-EDS/EELS is largely^46–48^ incompatible with direct investigation of (photo)­electrochemically active solid-liquid interfaces. Further, challenges arise when attempting to correlate compositional information derived from these near-surface sensitive techniques to the electrochemically active surface, particularly in the presence of near-surface compositional heterogeneity.

Here, we investigate the (photo)­electrocatalytic implications of near-surface compositional heterogeneity on alkaline OER activity, using mechanical strain to vary the Ni site density observed on TiO_2_ thin films grown by air annealing the superelastic alloy Nitinol (TiO_2_/NiTi). Application of tensile mechanical strain via tensilometer causes mechanical failure (cracking) of the TiO_2_ overlayer, manifesting in increased Ni site density that correlates with (photo)­electrochemical OER performance. Absent the sharp Ni^3+^/Ni^2+^ redox features used to electrochemically quantify surface Ni site density, however, the surface Ni composition would be inflated when measured by near-surface-sensitive characterization techniques due to the complex compositional heterogeneity observed throughout the near-surface of TiO_2_/NiTi. Large, ∼2 orders of magnitude, differences in Ni composition are observed on unstrained TiO_2_/NiTi when contrasting compositional information derived from surface-sensitive (electrochemical, typically ∼10^12^ Ni/cm^2^_geo_) and near-surface- or bulk-sensitive spectroscopic and spectrometric probes (TOF-SIMS, XPS, STEM-EDS, SEM-EDS, typically >10^14^ Ni/cm^2^). These results illustrate the sensitivity of reaction kinetics to surface composition and how linking the performance and intrinsic properties of catalytic surfaces remains a challenging endeavor, particularly when considering the complex morphologies and compositions of electrocatalysts under operating conditions. Further efforts are needed in developing *in situ* techniques to accurately characterize the composition and number of electrocatalytic active sites, while *ex situ* characterization techniques should be interpreted cautiously and result in physical models of surfaces that are self-consistent across multiple near-surface compositional probes.

## Methods

### Preparation of Rutile TiO_2_ Thin Films

Films of rutile TiO_2_ were thermally grown on polished, cleaned, and acid-etched polycrystalline foils of Nitinol (NiTi, Thermo Scientific). As-purchased NiTi foils were polished with a series of sequentially smaller particle size alumina powder slurries (5 μm, 0.3 μm, and 0.05 μm) and rinsed in water between each polishing step. All water used in this work was of millipure quality (i.e. >18 MΩ cm). Polished foils were then sequentially rinsed and sonicated for 10 min each in isopropanol (HPLC grade, Fisher Chemical), ethanol (anhydrous, Koptec), and water. Polished and cleaned foils were acid etched in a 1:4 hydrochloric acid (HCl, ACS grade, J.T. Baker) in water solution for 5 min to dissolve surface Ni sites before further rinsing and sonicating for 10 min in water. Polished, cleaned, and etched NiTi foils were then annealed in air at 500 °C for 30 min to form a thin film^49^ of predominantly TiO_2_ (∼60 nm thick by spectroscopic ellipsometry), which received an additional acid etch and water rinse and sonication step to remove residual surface Ni. Inductively coupled plasma mass spectrometry (ICP-MS, Agilent 8900 ICP-QQQ) indicated approximately an order of magnitude more Ni was dissolved in the acid etching step before formation of the TiO_2_ overlayer (∼0.6 μg Ni/mL) than after (∼0.07 μg Ni/mL).

### (Photo)­electrochemistry

All electrochemical measurements were performed in air-saturated 0.1 M NaOH (≥99.99%, BeanTown Chemical) using a Pt wire (99.9%, Thermoscientific) counter electrode and Ag/AgCl (CHI111, saturated KCl, ACS grade ≥99%, Sigma-Aldrich) reference electrode mounted in a separate fritted bridge tube to mitigate chloride ion contamination (Ametek K0065). Electrochemical potentials were calibrated against the reversible hydrogen electrode (RHE) by measuring the voltage difference against a master standard Ag/AgCl reference stored exclusively in saturated KCl (CHI111, saturated KCl, 0.197 V_SHE_)^50^ and corrected for pH assuming 58.16 mV/pH at 20 °C. Voltage drop corresponding to resistive losses (iR_S_ losses) were 100% corrected by treating the low-frequency linear intercept of a potentio- electrochemical impedance spectroscopy (PEIS) Nyquist plot as solution and contact resistance (R_S_). PEIS measurements were performed with 10 mV sinusoidal oscillations from 200 kHz to 100 mHz at 6 points per decade after holding potential at a non-Faradaic potential (typically 1 V_RHE_) for 1 min. All potentiostats used were manufactured by BioLogic (SP-300 or SP-240).

Working electrodes were mechanically strained using a 450 N capacity MTI/Fullam SEMTester controlled using MTESTQuattro software at a rate of 0.25 mm/min. Strained electrodes were clamped in custom stainless steel sample holders to retain the mechanically-applied strain. Strained working electrodes were back contacted using Cu tape connected to the stainless-steel sample holder, then mounted with a 5/32” inner diameter Viton gasket (Grainger) to a custom-made PEEK (photo)­electrochemical cell with quartz window (ThorLabs, Infrasil UDP105) mounted normal approximately 0.89” from the sample surface.^49^ All (photo)­electrochemical surface areas were reported on a geometric basis assuming the O-ring inner diameter defines the exposed electrode surface area (0.019 in^2^ = 0.124 cm^2^). Electrochemical cells and glassware were cleaned in a 1:4 hydrochloric acid in water solution for several hours before being thoroughly rinsed with water, stored overnight in water, and thoroughly rinsed and dried in nitrogen once more before use.

Broadband (photo)­electrochemical measurements were performed by exposing TiO_2_/NiTi samples to AM 1.5G light^51^ from a PICO KLMNO–DIR multi-LED lightsource (spectrum in Figure S1) and calibrated to 100 mW/cm^2^ using an externally calibrated Si photodiode calibrated at NREL. Incident photon to current efficiency measurements were performed by underfilling the photoelectrode area with a ∼2 mm diameter monochromated 300 W Xe arc lamp from 280 to 450 nm wavelength in 10 nm increments,^52^ the power of which was quantified using a commercial Si photodiode (Hamamatsu, S1336-8BQ) calibrated at NREL.

### Characterization

Inductively coupled plasma mass spectrometry (ICP-MS) was performed with an Agilent 8900 ICP-QQQ triple quadrupole instrument. Calibration curve standards ranging from 1-1200 ng/mL were prepared from a High Purity Standards calibration standard (ICP-MSCS-PE3-A-100, 10 μg/mL) diluted with ∼2% nitric acid (made from 67-70% Optima Grade Fischer Scientific diluted in Milli-Q water ≥18 MΩ cm). R values for the calibration curves were ≥0.9999 with detection limits of 0.2 ng/mL or better. The samples, including a blank, were prepared by diluting the etchant solutions 10x in a 2% nitric acid matrix. Measurements were taken in He mode (collision cell, 4.5 mL/min flow rate) and in O_2_ mode (reaction cell, He 1 mL/min, O_2_ 30%). An internal standard (ISTD) of ∼50 ppb of Y in 2% nitric acid was used. Nickel was monitored for m/z = 60 in He mode in single quad mode with Y 89 as the ISTD. The triple quad capability was used in O_2_ mode to monitor 60 to >60 (no mass shift) and 60 to >76 (mass shift) using Y 89 to >105 as the ISTD signal. The ISTD recovery in He mode was <80% for the samples precluding reportable data. In the O_2_ mode, the ISTD recovery was ∼90%. Titanium was monitored in “semiquant” mode as HF-containing calibration standards were avoided. Measurement method was single point, 100 sweeps, 3 replicates, 0.5 s integration time. The sample uptake time was 100 s to ensure stabilization of the signal.

X-ray diffraction (XRD) patterns were collected with a Bruker D8 Discover equipped with a Cu Kα X-ray source (1.5406 Å) and two-dimensional area detector (2D-XRD) that maps diffraction patterns in both 2θ and *χ*. 2D-XRD data were numerically integrated over *χ* along the range of 2θ values denoted in the main text. Diffraction features (diffraction angle and full-width at half maximum (FWHM)) were parameterized by subtracting a polynomial background and fitting each feature with a Voigt function (red lines in Figure S2).

Optical properties were measured from 200 to 1800 nm wavelength using a Woollam M-2000 variable angle spectroscopic ellipsometer (J.A. Woollam) with incident and reflected light between 64° and 84° from surface normal in 5° increments. Optical constants (n, k) of the TiO_2_ overlayer were modelled using CompleteEASE software (J.A. Woollam) by treating the substrate as a bulk NiTi sample (measured separately, Figure S3) and the TiO_2_ overlayer with a Kramers-Kronig consistent B-spline model using a Cauchy model to inform thickness and refractive indices in the non-absorbing region (>450 nm).

Surface-normal SEM was collected using a Hitachi S-4800 Scanning Electron Microscope operated at 3.0 kV, and surface normal EDS was collected on the same instrument with an integrated Thermo Scientific 4443C-3UPS-SN EDS detector operated at 20 kV. Cross sections for SEM and STEM analysis were prepared by depositing a 250 nm Pt and ∼3 μm W cap over an area of the TiO_2_/NiTi to protect the surface from focused-ion-beam (FIB) milling damage. FIB trenching was then performed at 30 kV and 10 μA with subsequent cross-sectional face cleaning at 30 kV and 1 μA for cross-sectional SEM-EDS analysis. Lamella for STEM-EDS analysis were contacted with a nanoprobe, FIB trenched at 30 kV and 10 μA, attached to a Mo TEM grid, and further polished with sequentially lower ion voltages from 30 down to 2 kV. Scanning transmission electron microscopy (STEM) imaging was performed on an aberration-corrected Themo Fisher Spectra 200 S/TEM, operated at 200 kV with a 24.2 mrad convergence angle. A parabolic background correction and Brown-Powell ionization cross-section model was used in STEM-EDS atomic fraction quantification.

Time of flight secondary ion mass spectrometry (TOF-SIMS) was performed using an ION-TOF TOF-SIMS V instrument. Analysis was completed by utilizing a 30 keV Bi^+^ primary ion beam (1 pA pulsed current) rastered over a 50 μm x 50 μm area. Sputtering was completed with a 3 keV Cs^+^ beam (25 nA current) rastered over a 150 μm x 150 μm area. After profiling, the crater depth was measured to correlate the sputter time scale to depth. Instrumental relative sensitivity factors for Ni and Ti were collected by averaging the first 10 nm of Ni^+^ and ^50^Ti^+^ ion intensities for a NiTi sample of known composition (Ni_0.52_Ti_0.48_, measured by normal-incidence SEM-EDS). Compositional errors of approximately 5% correspond to differences in matrix effects for TiO_2_/NiTi and deviations in the Ni:Ti ratio.

Laboratory-based X-ray photoelectron spectroscopy (XPS) data were obtained on a PHI VersaProbe III instrument using Al Kα radiation (1486.7 eV). The XPS data were calibrated with Au and/or Cu metal, which was cleaned via Ar-ion sputtering. The raw atomic concentration has a 5% error due to surface inhomogeneities, surface roughness, literature sensitivity values for peak integration, *etc.* Ambient pressure XPS (AP-XPS) measurements were performed at beamline 9.3.2 of the Advanced Light Source (ALS) at Lawrence Berkeley National Laboratory (LBNL).^53^ Details of sample mounting and data collection are provided in the SI and detailed elsewhere.^7,54,55^ Briefly, samples were mounted onto a ceramic button heater with a Pt heating element and electronically grounded to the analyzer by an Ir thermocouple pinned to the sample surface beneath a Au foil (used for binding energy calibration) and alumina chips to isolate the sample from the heating element. All binding energies were calibrated against the Au 4*f*_7/2_ feature at 84.0 eV. Samples were cleaned in situ by heating between 25 and 250 °C and exposing to 200 mTorr oxygen (Praxair, >99.999%). Spectra were collected with a 100 eV analyzer pass energy at 0.2 s/step and a 100 meV step size for core level or 1 eV step size for survey spectra.

## Results

### Physical Characterization and Manifestation of Mechanically Applied Strain

We begin by characterizing the physical manifestations of mechanically applied tensile strain (strain) on the predominantly rutile TiO_2_ thin films. TiO_2_ thin films are grown by air annealing polished and cleaned NiTi foils at 500 °C for 30 min (Figure 1a), with hydrochloric acid (∼10% HCl aqueous solution) etching steps before and after annealing to remove as many surface Ni sites as possible. Ellipsometry identifies a ∼60 nm thick TiO_2_ layer over the bare NiTi substrate (Figure S3). X-ray diffraction (XRD) reveals the complex, strain-dependent behavior of the TiO_2_/NiTi heterostructure (Figure 1b-d). Diffraction patterns for polished and cleaned NiTi substrates transform from a body-centered cubic (bcc) CsCl structure^56^ at low strain to a monoclinic martensite^57,58^ phase at higher strain (Figure S4).^59–62^ Upon formation of the TiO_2_/NiTi heterostructure via thermal oxidation (Figure 1a), two distinct diffraction peaks corresponding to rutile TiO_2_ appear at ∼27.4° (110) and ∼65.5° 2θ (310).^63,64^ The TiO_2_ (310) peak shifts to a higher angle and broadens as a function of applied strain (Figure 1b, dashed red arrows), in concert with a corresponding bcc NiTi (210) peak having similar interplanar spacing (∼70.5° 2θ). Indeed, the TiO_2_ (310) and NiTi (210) diffraction angles increase linearly between 0 and 5% strain and remain invariant for >5% strain (Figure 1c), when quantified by fitted Voigt functions (Methods). Changes in full-width at half-maximum (ΔFWHM) relative to 0% strain similarly indicate strain-induced broadening for both TiO_2_ (310) and NiTi (210) (Figure 1d), though the TiO_2_ (310) feature broadens to a greater extent. In contrast, the TiO_2_ (110) plane spacing and breadth appear invariant across the range of applied strain considered here (0-7%).

**Figure 1:**
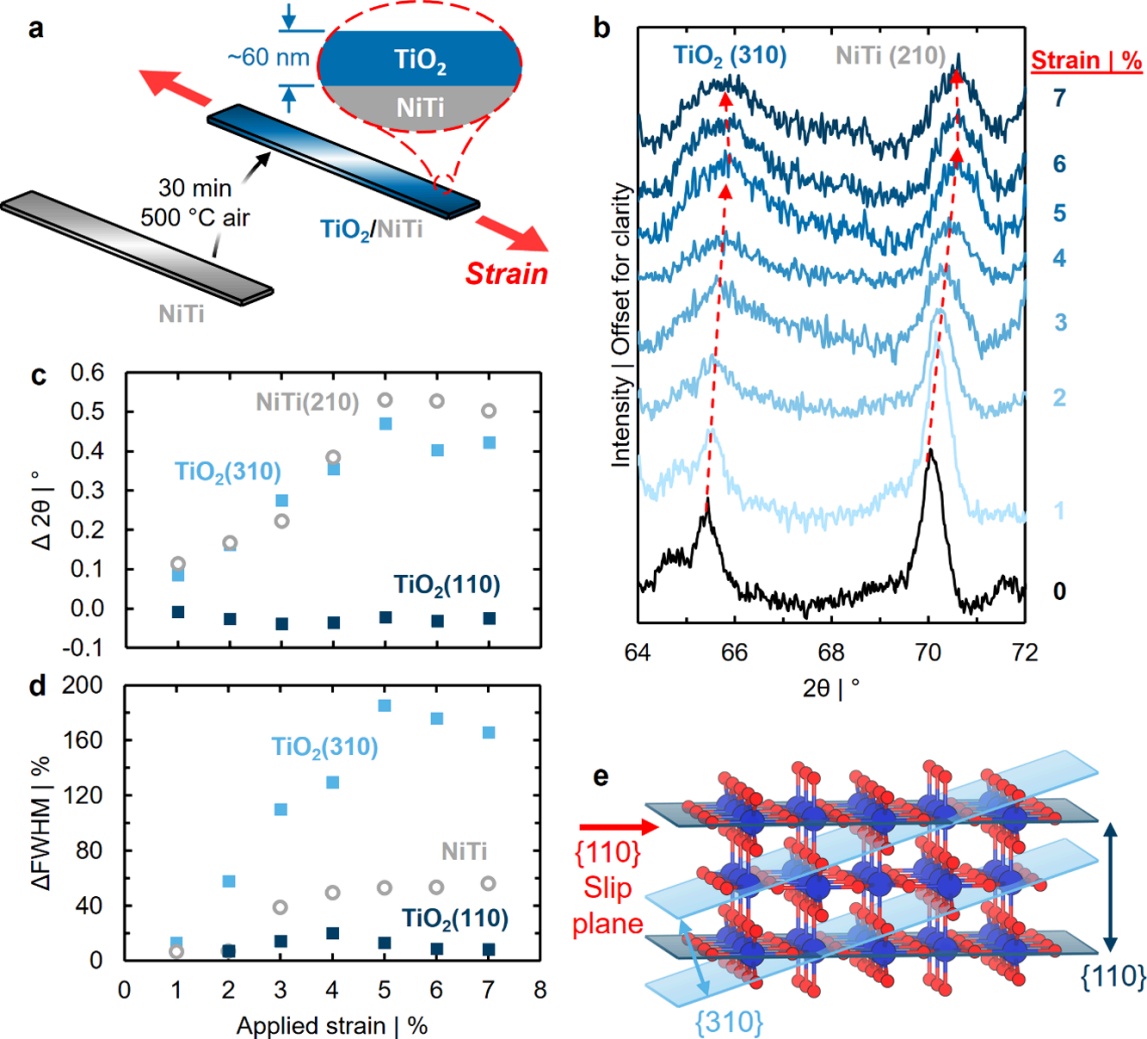
(a) Schematic illustration of a TiO_2_ thin film (blue) growth over a polished and cleaned NiTi substrate (gray) by thermal annealing at 500 °C in air for 30 min and the application of mechanical strain (red arrows and text). (b) XRD of the rutile TiO_2_ (310) and bcc NiTi (210) features as a function of increasing strain. Red dashed lines are a guide for the eye, denoting the qualitative trends in diffraction peak angles with increasing strain. The feature at ∼64.8° 2θ attenuates with increasing strain, suggesting it corresponds to one of the NiTi bcc planes. Quantified change in (c) peak diffraction angles and (d) FWHM as a function of applied strain relative to the unstrained state of the rutile TiO_2_ (110) (closed dark blue squares) and (310) (closed light blue squares) and bcc NiTi (210) (open gray circles). (e) Illustration of the rutile TiO_2_ crystal structure, indicating the (110) (dark blue) and (310) (light blue) diffraction planes detected by XRD, and the {110} slip plane (red arrow and text).^65–67^

Differences in strain-dependent behavior of TiO_2_ (110) and (310) planes may be explained by activation of a {110} slip plane (red arrow, Figure 1e),^65–67^ resulting in the observed compression (increasing 2θ) of the rutile TiO_2_ (310) plane without changing the (110) plane spacing. The FWHM of the TiO_2_ (310) and NiTi (210) planes increase with increasing strain, indicating a broadening distribution of lattice spacings caused by either decreasing crystallite size or a heterogeneous slip plane distribution throughout the TiO_2_ overlayer. The lack of a similar increase in FWHM of the rutile (110) peak (Figure S2) suggests that changes in crystallite size are not responsible. Rather, we suggest spatial heterogeneity in slip plane distribution is responsible, where the distribution of discrete slip planes varies across the film surface. Correspondingly, cracks are observed forming and spreading across the sample surface with increasing strain by visual observation and SEM (Figures S5 and S6), suggesting that activation of {110} slip planes leads to irreversible mechanical failure of the TiO_2_ crystal.

### (Photo)­electrochemical Manifestations: Strain or Substrate Effects?

Having identified how strain manifests physically in TiO_2_/NiTi, we next consider the (photo)­electrochemical implications of mechanically applied strain. As TiO_2_ is a well-established n-type semiconductor,^68^ photoelectrochemical probes may selectively provide insight into the influence of strain on OER performance and the physicochemical properties of TiO_2_. Indeed, despite the unusual architecture employing a ∼60 nm thin film semiconductor over a bulk metal, unstrained TiO_2_/NiTi demonstrates n-type semiconducting properties under AM 1.5G illumination. Open circuit potential shifts cathodically to ca. 0.2 V_RHE_ under illumination as the increased population of valence band holes drives down the minority carrier quasi-Fermi level (Figure S7). Cyclic voltammetric (CV) profiles similarly demonstrate unusual n-type behavior under AM 1.5G illumination (Figure 2a), appearing as a linear combination of dark and illuminated current-voltage responses. Photocurrent onset (ca. 1 V_RHE_) occurs at potentials less anodic than the OER equilibrium potential (1.23 V_RHE_). Light-limited photocurrents occur at potentials anodic of OER equilibrium and prior to the exponential current onset at ca. 1.6 V_RHE_ that corresponds to dark OER current (dashed black line). Photocurrent onset shifts toward less anodic potential as strain increases toward 1%, while illuminated open circuit potential remains nominally invariant (Figure S8). Short circuit current densities (measured at OER equilibrium potential) rise as strain increases from 0 to 1%. Similar observations are made when comparing current-voltage responses under steady-state chronoamperometric conditions (Figure S9).

**Figure 2:**
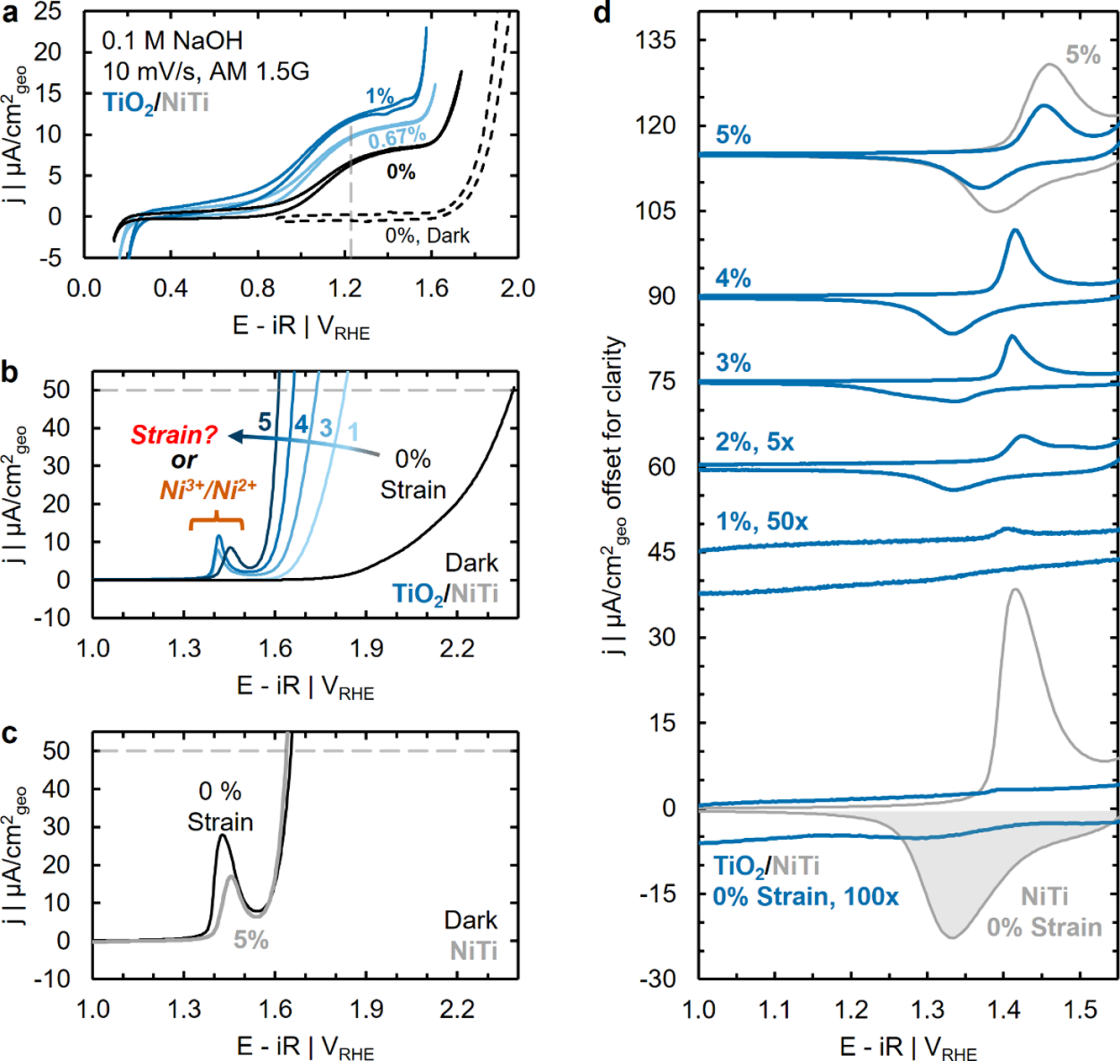
(a) Cyclic voltammetry of unstrained TiO_2_/NiTi in the dark (dashed black lines, 0% Dark) and under AM 1.5G illumination (solid lines) with increasing strain from 0% (black), 0.67% (light blue), and 1% (dark blue). Vertical gray dashed line denotes the OER equilibrium potential. (b,c) Anodic-going sweep of reproducible dark cyclic voltammograms for (b) unstrained TiO_2_/NiTi (black line and text, 0%) or under 1, 3, 4, and 5% strain (increasingly dark shades of blue) and (c) NiTi unstrained (thin black line and text, 0%) and strained to 5% (bold gray line and text). The horizontal dashed gray line denotes 50 μA/cm^2^_geo_ used to assess overpotential. The orange bracket in (b) denotes the range of literature potentials for the anodic-going voltammetric wave of pure and Fe-containing Ni oxyhydroxides, reported as the average plus and minus two standard deviations (Table S1).^18–21,69–75^ (d) Cyclic voltammograms highlighting the Ni^3+^/Ni^2+^ redox region for TiO_2_/NiTi (bold shades of blue) or NiTi (thin gray lines) as a function of applied strain (denoted). The current density axis is multiplied by a factor of 100 (0%), 50 (1%) and 5 (2%) for TiO_2_/NiTi voltammograms to enhance visibility of the Ni^3+^/Ni^2+^ redox features. The gray shading for 0% NiTi illustrates the area under the cathodic-going sweep used to determine the areal density of Ni sites (Ni/cm^2^_geo_).^21,76,77^ All electrochemical data are collected in 0.1 M NaOH at 10 mV/s and reported as the 3^rd^ cycle at 10 mV/s after having first been cycled 20 times at 50 mV/s.

Changes to the photoelectrochemical behavior of mechanically strained TiO_2_/NiTi suggest corresponding changes to one or all of the underlying photoabsorption, bulk charge transport, and kinetic properties.^5^ Measurements of incident-photon to current efficiency (IPCE) indicate nominally strain-independent performance (Figure S10), with ellipsometry indicating similarly strain-invariant absorption coefficient and refractive index (Figure S3). Similarly, two-point probe measurements of resistivity do not identify correlations between mechanically applied strain and bulk charge transport properties (Figure S11). The absence of correlations between mechanically-applied strain and bulk optoelectronic properties indicate that the intrinsic charge generation and transport properties of TiO_2_ are not responsible for the observed changes in the illuminated OER performance of TiO_2_/NiTi. Rather, variations in electrocatalytic kinetics are likely the cause, where non-illuminated (“dark”) measurements may provide greater insight.

Activity for dark OER nominally increases as TiO_2_/NiTi is increasingly strained from 0 to 5% (Figure 2b), mirroring trends observed under AM 1.5G illumination and in previous investigations of strained TiO_2_/NiTi architectures.^78^ Under 0% strain, >1000 mV of overpotential (2.4 V_RHE_) is required to generate a modest 50 μA/cm^2^_geo_. However, the overpotential required to achieve the same current density decreases to as little as 370 mV as strain is increased toward 5%. In contrast, bare NiTi substrates demonstrate strain-independent OER performance (Figure 2c), consistently requiring 410 ± 10 mV overpotential to generate 50 μA/cm^2^_geo_ of OER current from 0 to 5% strain. Overpotentials are chosen at low current densities (50 μA/cm^2^_geo_) to avoid the inherent mass transfer limitations caused by the narrow aperture used in this work (∼4 mm diameter).

While strain initially appears to modulate the OER kinetics of TiO_2_/NiTi, the sharp redox features at ca. 1.45 V_RHE_ correspond to the oxidation of Ni­(OH)_2_ and reduction of NiOOH (Ni^3+^/Ni^2+^, orange bracket in Figure 2b), where nickelates have long been recognized as highly active OER catalysts.^18,19,79–81^ The presence of Ni^3+^/Ni^2+^ redox features convolutes determination of the role of mechanically applied strain in influencing OER kinetics, as the overpotential requirements of TiO_2_/NiTi appear to asymptotically approach those of NiTi with increasing strain (Figure S12).

Indeed, the magnitude of Ni^3+^/Ni^2+^ feature intensity observed on TiO_2_/NiTi qualitatively trends with mechanically applied strain (Figure 2d). Very faint Ni^3+^/Ni^2+^ redox features are observed between 0 and 2% strain for TiO_2_/NiTi (bold blue lines; note current-axis magnification applied to TiO_2_/NiTi data). However, upon increasing to ≥3% strain, the magnitude of Ni^3+^/Ni^2+^ redox features in TiO_2_/NiTi becomes comparable to that observed on bare NiTi (thin gray lines). In contrast, the magnitude of the Ni^3+^/Ni^2+^ redox feature is fairly consistent on the bare NiTi substrate with increasing strain (Figure S13). In line with prior literature,^21^ the Ni^3+^/Ni^2+^ redox feature magnitude grows and shifts slightly in potential with electrochemical cycling (Figures S14 and S15). We therefore report the 3^rd^ voltammetric sweep collected at 10 mV/s after having first collected 20 sweeps at 50 mV/s.

As a means to deconvolute the effects of mechanically applied strain from the presence of Ni sites on the OER kinetics of TiO_2_/NiTi, we quantify the areal density of electrochemically available Ni sites (Ni site density, Ni/cm^2^_geo_) by integrating the area under the cathodic-going Ni^3+^/Ni^2+^ redox feature and assuming one electron per Ni site with unity Faradaic efficiency (Figure 2d, transparent gray region of 0% NiTi).^21,76,77^ This derivation of electrochemically available Ni site density is distinct from conventional definitions of capacitance-derived active surface area measurements^80,81^ (see Figure 3 and discussion in text) in that it exclusively accounts for contributions from Ni sites able to undergo redox processes (i.e. originating at the electrode surface), neglecting the surface area from TiO_2_. Reflecting the strain-dependent increase in Ni^3+^/Ni^2+^ feature intensity on TiO_2_/NiTi, Ni site density increases exponentially with applied strain from ∼1 × 10^12^ Ni/cm^2^_geo_ at 0% strain to ∼5 × 10^14^ Ni/cm^2^_geo_ by 3% strain (Figure S16). As strain increases to ≥3%, Ni site density on TiO_2_/NiTi coincides with that observed on the strain-independent NiTi (1.1 × 10^15^ ± 0.5 × 10^15^ Ni/cm^2^_geo_). We note that Ni^3+^/Ni^2+^ redox feature magnitude decreases slightly with increasing strain for NiTi (Figure S16), corresponding to a lower areal density of Ni sites caused by the strained, monoclinic phase of NiTi having a larger average lattice constant (0.3715 nm)^57,58^ than the unstrained, bcc phase (0.3015 nm).^56^

**Figure 3:**
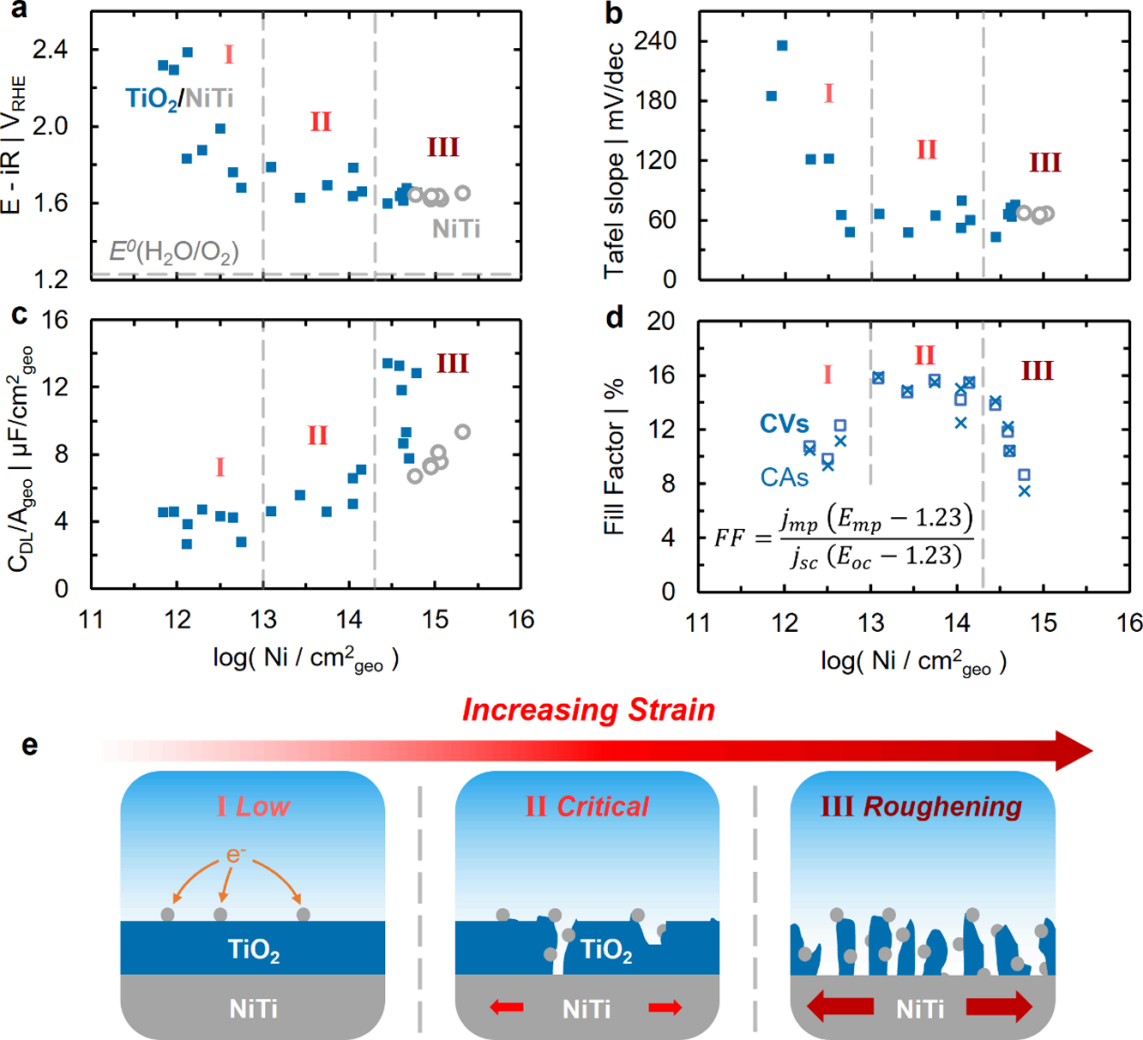
Influence of electrochemically available Ni site density on (a) overpotential required to generate 50 μA/cm^2^_geo_ of OER current, (b) Tafel slope, and (c) double layer capacitance normalized by geometric surface area (C_DL_/A_geo_) for TiO_2_/NiTi (closed blue squares) and NiTi (open gray circles) measured in 0.1 M NaOH in the dark. (a) Horizontal gray dashed line denotes OER equilibrium potential. (d) Fill factor of TiO_2_/NiTi measured under AM 1.5G illumination by both cyclic voltammetry (CVs, open squares) and chronoamperometry (CAs, x’s). (a–d) Vertical gray dashed lines delineate approximate distinctions between the (I) low (<10^13^ Ni/cm^2^_geo_, pink text), (II) critical (10^13^ to 2 × 10^14^ Ni/cm^2^_geo_, red text), and (III) roughening regimes (>2 × 10^14^ Ni/cm^2^_geo_, dark red text) as illustrated in (e), where the TiO_2_ overlayer (dark blue) actively breaks apart with increasing strain to expose additional Ni sites and NiTi substrate (gray).

Having identified a correlation between Ni site density and mechanically applied strain, we next contextualize changes in the (photo)­electrochemical performance of TiO_2_/NiTi as a function of Ni site density in three distinct regimes (Figure 3). Electrochemical potentials required to generate 50 μA/cm^2^_geo_ of OER current decrease from ∼2.4 V_RHE_ to ∼1.6 V_RHE_ as Ni site density increases from ∼10^12^ to ∼10^13^ Ni/cm^2^_geo_ within the low Ni site density regime (Figure 3a, regime I). Similar trends are observed when decreasing the overpotential requirement to 15 μA/cm^2^_geo_, indicating that both current densities are kinetically limited (Figure S17). Decreased OER overpotentials are a manifestation of changes in observed Tafel kinetics, measured by chronoamperometry (Figure S18), as the Ni site density increases. Tafel slopes decrease from a sluggish ∼240 mV/dec—indicating poor OER kinetics of the predominantly TiO_2_ overlayer—to ∼60 mV/dec within the low Ni site density regime (Figure 3b, regime I). Tafel slopes of ∼60 to 70 mV/dec—consistent with deprotonation of adsorbed hydroxide as a rate-limiting step^82,83^ —have consistently been observed on bulk Ni^18,84^ and Ni­(OH)_2_ surfaces.^70^

OER overpotential and Tafel slopes for TiO_2_/NiTi approach values similar to those of the bare NiTi substrate as electrochemically available Ni site density exceeds 10^13^ Ni/cm^2^_geo_ (Figure 3, regimes II and III; overpotentials of ∼370 mV and Tafel slopes ∼60 mV/dec). Invariant Tafel slopes indicate the intrinsic kinetics of Ni sites dominate observed OER current, while unchanging overpotential suggests that additional Ni sites are nominally inactive. Curiously, this indicates that Ni site densities as low as 10^13^ Ni/cm^2^_geo_ (∼1% coverage, assuming a rutile^63^ TiO_2_ lattice averaging 1.0 × 10^15^ Ti atoms/cm^2^) are sufficient to obtain most of the kinetic benefits for the OER.

In contrast, double layer capacitance demonstrates the opposite trends, showing little change in the low Ni site density regime (4.5 ± 0.2 μF/cm^2^_geo_) before increasing to values similar to those of a bare NiTi substrate in the critical Ni site density regime (7.7 ± 0.9 μF/cm^2^_geo_, regimes I and II). Absent the presence of sharp Ni^3+^/Ni^2+^ redox features, changes in double layer capacitance alone would fail to capture the increased Ni site loading that influences OER kinetics in the low Ni site density regime. This highlights the limitations of relying exclusively on double layer capacitance to characterize electrochemically active surface area when surface compositional heterogeneity exists between samples.^80,81^ As Ni site density exceeds ∼2 × 10^14^ Ni/cm^2^_geo_ (Figure 3c, regime III), double layer capacitance continues to increase and overcomes the capacitance of the bare NiTi substrate in a sign of surface roughening. Roughening is likely caused by crack formation within the TiO_2_ overlayer with increasing strain, as observed optically (Figure S5) and by SEM (Figure S6).^49,78^

Similar to changes in dark electrochemical kinetics and capacitance, photoelectrochemical OER fill factors for TiO_2_/NiTi follow Ni-loading-dependent performance under AM 1.5G illumination (Figure 3d). Here, we use fill factor to assess photoelectrochemical performance:

$$FF|\%=\frac{P_{\mathrm{mp}}}{P_{\max}}=\frac{\left(E_{\mathrm{mp}}-1.23\left[V_{RHE}\right]\right)j_{\mathrm{mp}}}{\left(E_{\mathrm{oc}}-1.23\left[V_{RHE}\right]\right)j_{\mathrm{sc}}}\times 100\%$$ 1

where fill factor (*FF*) is defined as the maximum power density (*P*_mp_) achieved at an optimal potential (*E*_mp_) and current density (*j*_mp_) and normalized by the maximum theoretical power density (*P*_max_) obtainable by operating at open circuit potential (*E*_oc_) and short circuit current density (*j*_sc_). Strain-dependent power-voltage curves are provided in the Supporting Information to illustrate the potential dependence of *P*_mp_ and choice of *E*_mp_, *j*_mp_, *E*_oc_, and *j*_sc_ (Figure S19).

Within the low Ni site density regime (I), fill factors rise from ∼10% at ∼10^12^ Ni/cm^2^_geo_ to 15% by 10^13^ Ni/cm^2^_geo_. Stable fill factors of ∼15% are then achieved in the critical Ni site density regime (II) between 10^13^ and 2 × 10^14^ Ni/cm^2^_geo_, before falling in the roughening regime (III, >2 × 10^14^ Ni/cm^2^_geo_). Decreasing fill factors within the roughening regime supports our prior observation that surface roughening explains increased double layer capacitance (Figure 3c, III). Rougher TiO_2_/NiTi surfaces may provide fewer TiO_2_ sites per geometric area that are capable of generating electron-hole pairs (e.g., mechanical delamination of TiO_2_) or introduce a greater density of defective surface recombination centers. While an initial rise in fill factor is similarly observed at low strain, fill factor data at higher strain values become stochastic, illustrating that strain alone fails to explain the observed trends in photoelectrochemical performance (Figure S20).

Although mechanically-applied strain serves as the independent variable of this work, it fails to explain in a physically meaningful way the observed trends in (photo)­electrochemical kinetics and particularly double layer capacitance and fill factor (Figure S20). Kinetic parameters plateau above ∼2% strain, and double layer capacitance and fill factor respond stochastically with increasing strain. Rather, we attribute the staged rise in OER kinetics and double layer capacitance to strain-dependent mechanical failure of the TiO_2_ overlayer (cracking), manifesting in increased exposure of OER active Ni sites (Figure 3e). Under low-strain conditions (regime I), the TiO_2_ overlayer transitions from being nominally mechanically sound to having small fissures develop (Figures S5 and S6), where small amounts of Ni become exposed and eventually dominate OER performance. However, as strain increases further, additional fissures form to expose Ni sites that were previously buried within the near-surface region of the TiO_2_ overlayer (regime II). Under the highest strains considered here (and correspondingly the highest Ni site densities), complete failure of the TiO_2_ overlayer occurs (regime III), exposing the NiTi substrate and leading to a corresponding rise in double layer capacitance as the actual electrochemically active surface area of the TiO_2_/NiTi heterostructure increases.

### Information Depth and Its Role in Characterizing Surface Composition

The dependence of heterogeneously catalyzed reaction kinetics on intrinsic surface properties necessitates an understanding of active site composition. This is particularly important when catalyst architectures display widely variant surface composition, such as the ∼3 orders of magnitude change in Ni site density caused by application of mechanical strain to the TiO_2_/NiTi system considered here (Figure 3). Absent the sharp Ni^3+^/Ni^2+^ redox features observed on TiO_2_/NiTi, however, compositional probes would need to have sufficient detection limits and interface sensitivity to identify the presence of Ni sites (Figure S21) and whether detected Ni atoms are located at the electrochemically active surface or are buried within the inactive near-surface several nm. Bulk-sensitive probes are unable to capture near-surface accumulation of Ni-containing species, being limited by either information depth (SEM-EDS and Raman, Figures S22 and S23) or detection modes (Raman, Figure S23). Rather, we contrast the Ni site density of unstrained TiO_2_/NiTi detected at the surface (measured electrochemically at ∼10^12^ Ni/cm^2^_geo_) against that detected using near-surface sensitive probes, identifying challenges posed by compositional heterogeneity when attempting to correlate (photo)­electrochemical kinetics and surface composition observed at these two distinct length scales (Figure 4a).

**Figure 4:**
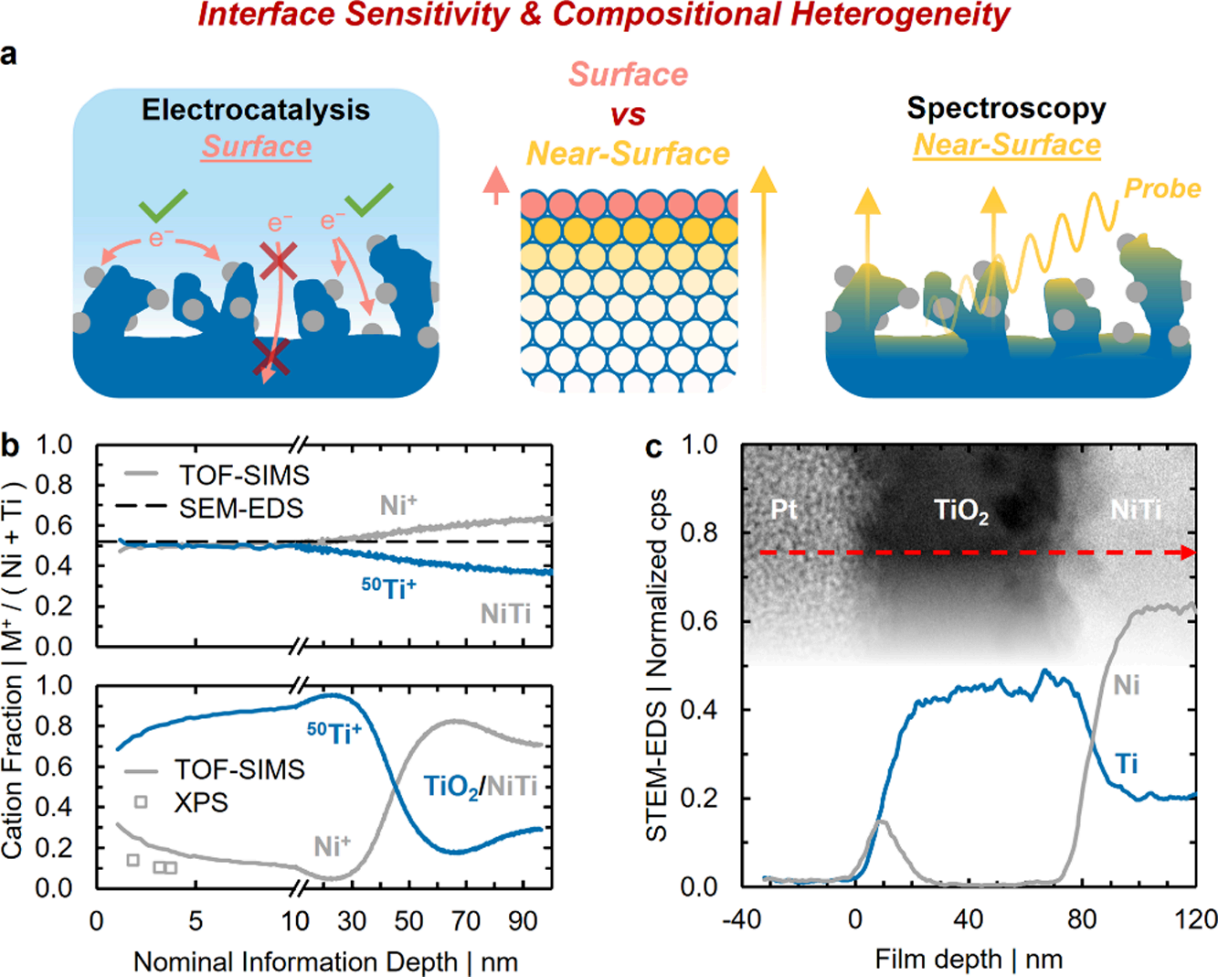
(a) Schematic illustration of the challenges posed when comparing compositional information derived from surface*-* (left, i.e. charge transfer across the atomic-scale solid-liquid interface) and near-surface-sensitive probes (right, i.e. spectroscopic and spectrometric probes) in the presence of near-surface compositional heterogeneity (Ni shown as gray spheres in the blue TiO_2_ matrix). Center panel provides a simplified illustration of the concept of surface (pink fill) vs near-surface (faded yellow fill) sensitivity, where information depth is illustrated by the corresponding arrows. (b) TOF-SIMS comparison of the cation composition of Ni (Ni^+^, gray line) and Ti (^50^Ti^+^, blue line) for (top) a bare NiTi substrate and (bottom) TiO_2_/NiTi as a function of sputter depth. The Ni cation fraction for NiTi is compared against the bulk-sensitive SEM-EDS Ni composition (top, black dashed lines), while the TiO_2_/NiTi Ni cation fraction is compared against near-surface sensitive synchrotron-based XPS (bottom, open gray squares). (c) STEM HAADF image and EDS line scan of a cross-section of the TiO_2_/NiTi interface with a Pt capping layer to protect the TiO_2_ surface during focused ion beam liftout. The red dashed line indicates the origin and direction of the line scan used to map Ni and Ti intensities through the depth of the TiO_2_/NiTi heterostructure.

We begin by discussing the sputtering depth dependent ratio of Ni to total metal cation signal measured by TOF-SIMS (i.e., Ni/(Ni + Ti)), contrasting the observed compositional homogeneity of NiTi against the compositional heterogeneity of TiO_2_/NiTi. TOF-SIMS of a polished and cleaned NiTi surface identifies an equimolar composition of Ni^+^ and ^50^Ti^+^ ions within the first ∼10 nm (Figure 4b, top). Equimolar near-surface composition agrees with bulk composition determined by normal incidence SEM-EDS measurements (black dashed line), and largely mirrors the surface Ni composition detected electrochemically at 1.1 × 10^15^ Ni/cm^2^_geo_ (42% Ni, or 2.7 × 10^15^ atoms/cm^2^ assuming an unstrained bcc^56^ lattice). Sputtering artifacts cause TOF-SIMS-derived compositions to deviate at depths greater than the initial ∼10 nm,^85,86^ where differences in sputtering rates for Ni^+^ and ^50^Ti^+^ ions lead to the appearance of a Ni-rich bulk composition. While surface, near-surface, and bulk compositions are comparable for compositionally homogeneous (photo)­electrocatalyst architectures, this comparison becomes increasingly challenged when introducing compositional inhomogeneity within the near-surface.

Contrasting compositionally homogeneous NiTi, compositional heterogeneity is observed as a function of TOF-SIMS sputtering depth in unstrained TiO_2_/NiTi (Figure 4b, bottom). Counts for ^50^Ti^+^ are elevated in the predominantly TiO_2_ overlayer and decrease as the sputtering depth moves into the Ni^+^ rich NiTi bulk. Focusing on the TiO_2_-terminated near-surface region, however, TOF-SIMS identifies a ∼30% Ni/(Ni + Ti) ratio within the first several nm of the surface (∼3.0 × 10^14^ Ni/cm^2^_geo_ assuming Ni atoms displace Ti atoms in a rutile TiO_2_ lattice averaging 1.0 × 10^15^ Ti atoms/cm^2^),^63^ providing a stark contrast with the low Ni site density observed electrochemically (1.7 × 10^12^ ± 1.3 × 10^12^ Ni/cm^2^_geo_). Formation of near-surface Ni may be caused by exsolution and phase segregation, as Ni has a low (<3 at. %) solubility in TiO_2_.^87^ The Ni^+^ fraction decreases steadily within the surface-most ∼25 nm of the TiO_2_ thin film before rising sharply in the Ni^+^ rich NiTi bulk. The broad distribution of Ni^+^ throughout the TiO_2_ thin film may be due to a combination of surface roughness effectively extending the observed solid-gas interface into the bulk and other sputtering artifacts (e.g., non-equivalent rates for Ni^+^ and ^50^Ti^+^).^85,86^

Similar surface Ni enrichment is observed when depth profiling TiO_2_/NiTi with XPS by varying synchrotron-based incident photon energy (Figure 4b bottom, open gray squares). The Ni/(Ni + Ti) ratio rises from 10 to 14% (1.1 to 1.6 × 10^14^ Ni/cm^2^) as information depth^45^ is decreased from 3.9 to 1.9 nm in the predominantly TiO_2_ matrix by decreasing incident photon energy from 750 to 315 eV.^31,40,54,55^ The simple model used to extract these compositions assumes a compositionally homogeneous analysis region (see Figure S24 and discussion in Supporting Information), similar to that used in the software of lab-based XPS instruments. Physical models containing more complex compositional and morphological heterogeneity can be accessed with NIST’s SESSA software package^88,89^ to better emulate variations in Ni composition with probe depth observed here (Figure S25). Simple models incorporating only one form of heterogeneity (e.g., NiO islands, particles, buried layers) fail to capture the observed trend in Ni/(Ni + Ti) with information depth. However, by combining multiple morphologies of heterogeneity into a single model (NiO in both islands with buried layers), SESSA models are better able to emulate the observed spatial variance in Ni/(Ni + Ti) observed experimentally here (Figure S24).

STEM high-angle annular dark field (HAADF) images capture well the rough TiO_2_ surface and TiO_2_/NiTi interface (Figure 4c). Compositional heterogeneity in the corresponding STEM-EDS line scan agrees with that observed via sputtering and synchrotron-based XPS, where the NiTi bulk is covered by a ∼75 nm thick primarily TiO_2_ overlayer containing near-surface Ni enrichment. Similar depth-dependent dispersion of Ni is observed in cross-sectional EDS maps with a TiO_2_ region separating a Ni-enriched near-surface from a Ti-depleted NiTi bulk (Figure S26).

Compositional results obtained from near-surface sensitive probes contrast with the surface Ni composition of unstrained TiO_2_/NiTi obtained electrochemically by ∼2 orders of magnitude. While near-surface sensitive techniques can identify Ni accumulation in the near-surface region, it is challenging to identify the extent to which detected Ni exists at the electrochemically active surface. Information depth of TOF-SIMS, XPS, and STEM-EDS approach single nm under ideal circumstances (i.e. atomically smooth, compositionally homogeneous samples).^90^ However, the combination of surface roughness (broadened sputtering resolution and ill-defined interfaces) and compositional heterogeneity contribute to the uncertainty when attempting to identify whether Ni sites originate from the electrochemically active surface or deeper into the near-surface. Extending beyond the TiO_2_/NiTi architecture considered here, other systems lacking similarly surface-sensitive indicators of active site composition (e.g. voltammetric Ni^3+^/Ni^2+^ redox waves) may struggle to accurately identify the origins of (photo)­electrochemical activity.

## Conclusion

Here, we have presented a case study illustrating the challenge of ascribing (photo)­electrochemical performance to intrinsic surface properties in the face of near-surface heterogeneity. Mechanically-applied tensile strain tunes the electrochemically available Ni site density observed on a predominantly TiO_2_ thin film grown by air annealing the superelastic alloy NiTi. Application of strain to the TiO_2_/NiTi system activates {110} slip planes within rutile TiO_2_, leading to mechanical failure (cracking) of the TiO_2_ overlayer and increased exposure of Ni sites. OER kinetics trend in three distinct regimes with the areal density of Ni sites, quantified electrochemically by the sharp Ni^3+^/Ni^2+^ redox features observed in cyclic voltammetry. In the low Ni site density regime (∼10^12^ to 10^13^ Ni/cm^2^_geo_), OER overpotentials decrease by ∼800 mV as Tafel slopes shift from values indicating sluggish kinetics (∼240 mV/dec) toward more facile kinetics, comparable to a bare NiTi substrate (∼60 mV/dec). Kinetics are not enhanced further with increased Ni site density (>10^13^ Ni/cm^2^_geo_), though double layer capacitance rises to values comparable to the bare NiTi substrate before eventually growing further as the TiO_2_ overlayer forms cracks and roughens. Similar trends are observed in photoelectrochemical probes, where the fill factor trends with Ni site density: rising in the low regime, stabilizing at the critical regime, and decaying in the roughening regime as the TiO_2_ overlayer undergoes mechanical failure.

Absent the sharp Ni^3+^/Ni^2+^ redox features observed electrochemically, near-surface-sensitive probes of composition (TOF-SIMS, XPS, and STEM-EDS) suggest Ni/(Ni + Ti) ratios of ∼10–30% under unstrained conditions, as opposed to the <1% surface Ni composition detected electrochemically. TOF-SIMS, STEM-EDS, and synchrotron-based XPS clearly identify Ni accumulation within the near-surface region, though all techniques struggle to identify where in those several nm the Ni signal originates. Disparities in surface and near-surface sensitive composition further highlight the challenges associated with attributing the performance of applied electrochemical systems to assumed material properties. These results reinforce the importance and value of fundamental investigations of interfaces with well-controlled composition and crystalline structure and highlight the importance of interpreting surface characterization techniques with physically grounded and self-consistent models using several different near-surface sensitive probes.

## Supplementary Material



## Notes

Tabulated summary of Ni^3+^/Ni^2+^ redox feature potentials; additional figures for experimental XRD, (photo)­electrochemical, ellipsometry, two-point probe, SEM-EDS, Raman,^91–95^ and XPS data;^96–98^ photographic, SEM, and STEM images of TiO_2_/NiTi samples under varying amounts of strain; and spectral response of the broadband light source used in this study (PDF)

## ∇

Department of Chemical Engineering, IIT Guwahati, Guwahati 781039, Assam, India
