# Supplementary material for: Oxygen Evolution on Mechanically Strained TiO2/NiTi: Implications of Compositional Heterogeneity at (Photo)electrocatalytic Interfaces
Source: ACS Electrochem. 2025 Jul 31;1(10):2177–89. doi: 10.1021/acselectrochem.5c00228 (PMC12498402; doi:10.1021/acselectrochem.5c00228)
Supplement: Supplementary file 1 [file ec5c00228_si_001.pdf]

Supporting Information For:

Oxygen Evolution on Mechanically-Strained TiO_2/NiTi: Implications of Compositional Heterogeneity at (Photo)Electrocatalytic Interfaces

O. Quinn Carvalho,[1] Nikita S. Dutta,[1] Debjit Ghoshal,[1†] Steven P. Harvey,[1] Ross A. Kerner,[1] Shuya Li,[1] Zebulon G. Schichtl,[1] Patrick Walker,[1] Logan M. Wilder,[1] Gustavo Z. Girotto,[2,3] Maximilian Jaugstetter,[4] Slavomir Nemšák,[2,5] Ethan J. Crumlin,[2,4] Elisa M. Miller,[1]* Ann L. Greenaway[1]*

[1] Materials, Chemistry, & Computational Science Directorate, National Renewable Energy Laboratory, Golden, CO 80401, USA

[2] The Advanced Light Source, Lawrence Berkeley National Laboratory, Berkeley, CA 94705, USA

[3] Graduate Program in Physics, Federal University of Rio Grande do Sul, Porto Alegre, RS 90010-150, Brazil

[4] Chemical Sciences Division, Lawrence Berkeley National Laboratory, Berkeley, CA 94705, USA

[5] Department of Physics and Astronomy, University of California, Davis, Davis, CA 95616, USA

[†] Current address: Department of Chemical Engineering, IIT Guwahati, Guwahati 781039, Assam, India

* Corresponding email: Ann.Greenaway@nrel.gov, Elisa.Miller@nrel.gov

SI Table of Contents

Supporting Tables S2

Supporting Figures S3

Supporting XPS Figures and SESSA Analysis S21

Supporting Information References S26

Supporting Information

Supporting Tables

Table S1. Summary of literature values for Ni^{3+}/Ni^{2+} redox features.

<table>
<tr><th rowspan="2">Fe?^</th><th rowspan="2">x in $Fe_xNi_{1-x}OOH$</th><th rowspan="2">Electrolyte</th><th rowspan="2">ν | mV/s</th><th rowspan="2">Cycle #</th><th colspan="2">E_{peak}[a] | V_{RHE}</th><th rowspan="2">Ref.</th></tr>
<tr><th>Ni^{3+}/Ni^{2+}</th><th>Ni^{2+}/Ni^{3+}</th></tr>
<tr><td>N</td><td>N/A</td><td>0.1 M KOH</td><td>Not reported</td><td>Not reported</td><td>1.331</td><td>1.446</td><td>1</td></tr>
<tr><td>N</td><td>N/A</td><td>1 M KOH</td><td>20</td><td>1 hr @ 10 mA/cm^2</td><td>1.349</td><td>1.401</td><td>2</td></tr>
<tr><td>N</td><td>N/A</td><td>0.1 M KOH</td><td>1</td><td>Not reported</td><td>1.282</td><td>1.400</td><td>3</td></tr>
<tr><td>N</td><td>N/A</td><td>0.1 M KOH</td><td>10</td><td>5-10</td><td>1.288</td><td>1.345</td><td>4</td></tr>
<tr><td>N</td><td>N/A</td><td>1 M KOH</td><td>10</td><td>12</td><td>1.286</td><td>1.348</td><td>5</td></tr>
<tr><td>N</td><td>N/A</td><td>0.1 M NaOH</td><td>10</td><td>Not reported</td><td>1.328</td><td>1.410</td><td rowspan="2">6</td></tr>
<tr><td>N</td><td>N/A</td><td>1 M NaOH</td><td>10</td><td>Not reported</td><td>1.322</td><td>1.386</td></tr>
<tr><td>N</td><td>N/A</td><td>25 w% KOH</td><td>10</td><td>100</td><td>1.225</td><td>1.331</td><td>7</td></tr>
<tr><td>N</td><td>N/A</td><td>1 M KOH</td><td>10</td><td>1</td><td>1.287</td><td>1.359</td><td rowspan="2">8</td></tr>
<tr><td>N</td><td>N/A</td><td>1 M KOH</td><td>10</td><td>13</td><td>1.297</td><td>1.366</td></tr>
<tr><td>Y</td><td>Not measured</td><td>1 M KOH</td><td>20</td><td>1 hr @ 10 mA/cm^2</td><td>1.356</td><td>1.412</td><td>2</td></tr>
<tr><td>Y</td><td>0.1</td><td>0.1 M KOH</td><td>1</td><td>Not reported</td><td>1.364</td><td>1.472</td><td>3</td></tr>
<tr><td rowspan="8">Y</td><td>0.92</td><td rowspan="8">0.1 M KOH</td><td rowspan="8">10</td><td rowspan="8">5-10</td><td>1.464</td><td rowspan="8">Not measured</td><td rowspan="8">4</td></tr>
<tr><td>0.7</td><td>1.427</td></tr>
<tr><td>0.52</td><td>1.405</td></tr>
<tr><td>0.44</td><td>1.401</td></tr>
<tr><td>0.33</td><td>1.364</td></tr>
<tr><td>0.3</td><td>1.360</td></tr>
<tr><td>0.25</td><td>1.350</td></tr>
<tr><td>0.08</td><td>1.314</td></tr>
<tr><td>Y</td><td>0.1</td><td>25 w% KOH</td><td>10</td><td>100</td><td>1.281</td><td>1.367</td><td>7</td></tr>
<tr><td rowspan="2">Y</td><td>0.05</td><td rowspan="2">1 M KOH</td><td rowspan="2">10</td><td>1</td><td>1.311</td><td>1.380</td><td rowspan="2">8</td></tr>
<tr><td>0.05</td><td>13</td><td>1.315</td><td>1.401</td></tr>
<tr><td rowspan="3">Y</td><td rowspan="3">Not measured</td><td rowspan="3">0.1 M KOH</td><td rowspan="3">10</td><td>1</td><td rowspan="3">Not measured</td><td>1.431</td><td rowspan="3">9</td></tr>
<tr><td>15 min. 1.5 V_{RHE}</td><td>1.417</td></tr>
<tr><td>75 min. 1.5 V_{RHE}</td><td>1.416</td></tr>
<tr><td rowspan="2">Y</td><td rowspan="2">Not measured</td><td rowspan="2">0.5 M KOH</td><td rowspan="2">100</td><td>1</td><td>1.347</td><td>1.435</td><td rowspan="2">10</td></tr>
<tr><td>10</td><td>1.343</td><td>1.424</td></tr>
<tr><td rowspan="2">Y</td><td rowspan="2">Not measured</td><td rowspan="2">0.1 M NaOH</td><td rowspan="2">100</td><td>1</td><td>1.356</td><td>1.488</td><td rowspan="2">11</td></tr>
<tr><td>“Cycled”</td><td>1.351</td><td>1.477</td></tr>
</table>

^ Rows labelled “N” have attempted to purify electrolytes of Fe contamination, while “Y” denotes measurements where Fe was introduced either intentionally by fabricating an electrode where $x > 0$ for $Fe_xNi_{1-x}OOH$ or contains residual contaminant Fe.

[a] E_{peak} used to denoted the maximum (minimum) current measured for oxidation Ni^{2+}/Ni^{3+} (reduction Ni^{3+}/Ni^{2+}) features.

Supporting Figures

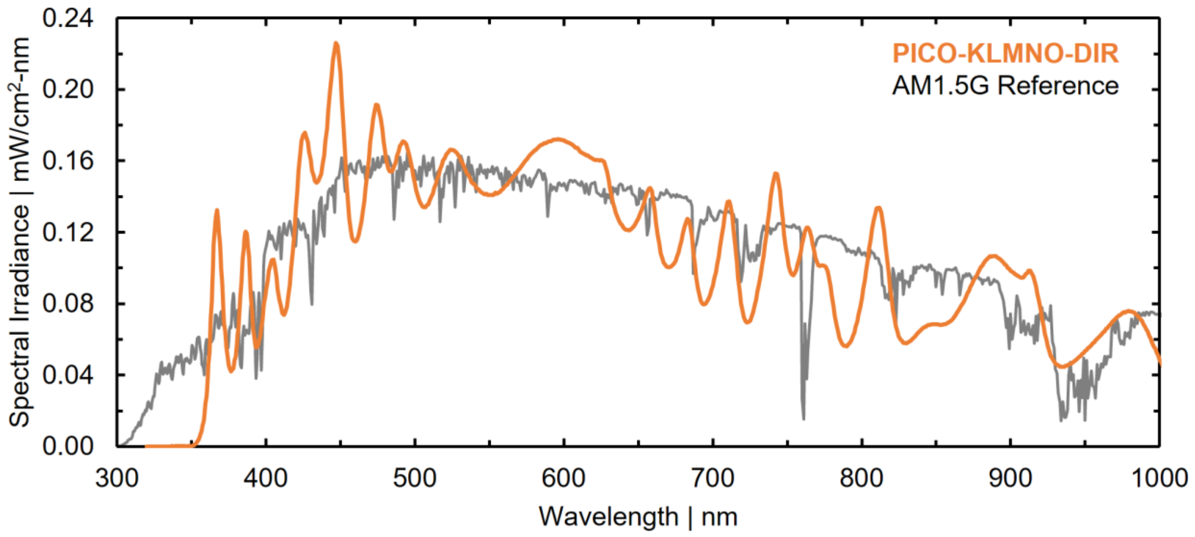

Figure S1. Spectral irradiance vs wavelength for the AM 1.5G spectrum[12] (grey) and the PICO-KLMNO-DIR (orange) multi-LED light source used in this study for broadband illumination.

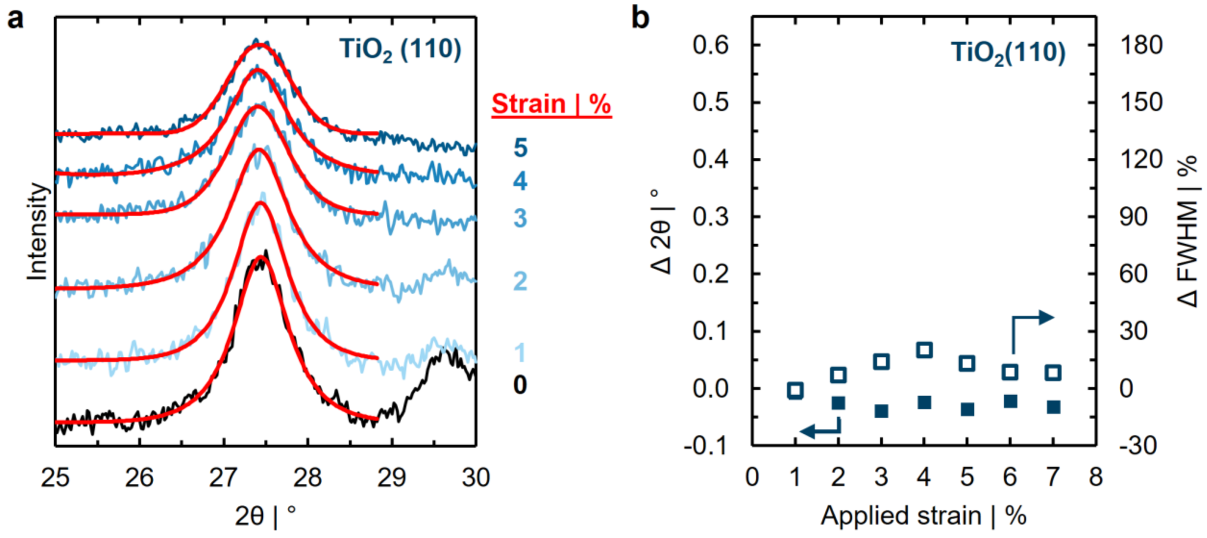

Figure S2. (a) High-resolution XRD of the rutile TiO_2 (110) peak as a function of increasing strain from 0 (black) to 7% (denoted with increasingly dark shades of blue lines) with corresponding Voigt function fittings to extract diffraction peak angles and FWHM shown as red lines. (b) Quantified changes in rutile (110) diffraction peak angle ($\Delta 2\theta$, closed dark blue squares, primary y-axis) and FWHM (Δ FWHM, open dark blue squares, secondary y-axis) as a function of applied strain.

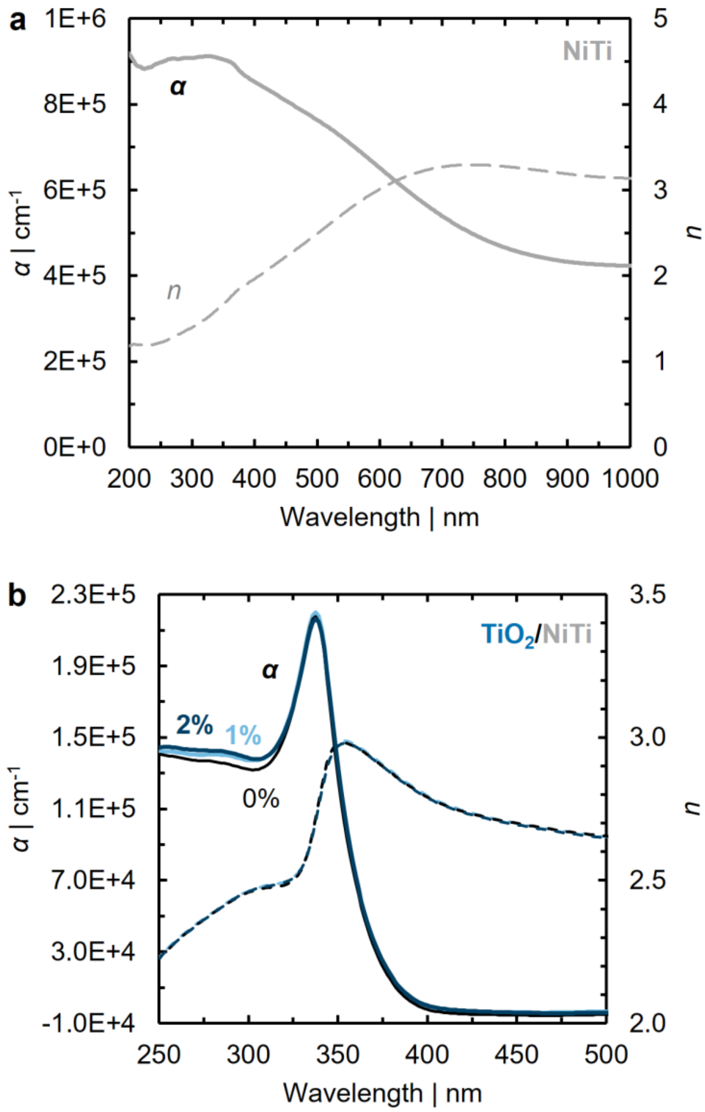

Figure S3. Wavelength-dependent absorption coefficient (α, bold solid lines) and index of refraction (n, thin dashed lines) for (a) NiTi and (b) TiO_2/NiTi strained to 0% (black), 1% (light blue) and 2% (dark blue lines).

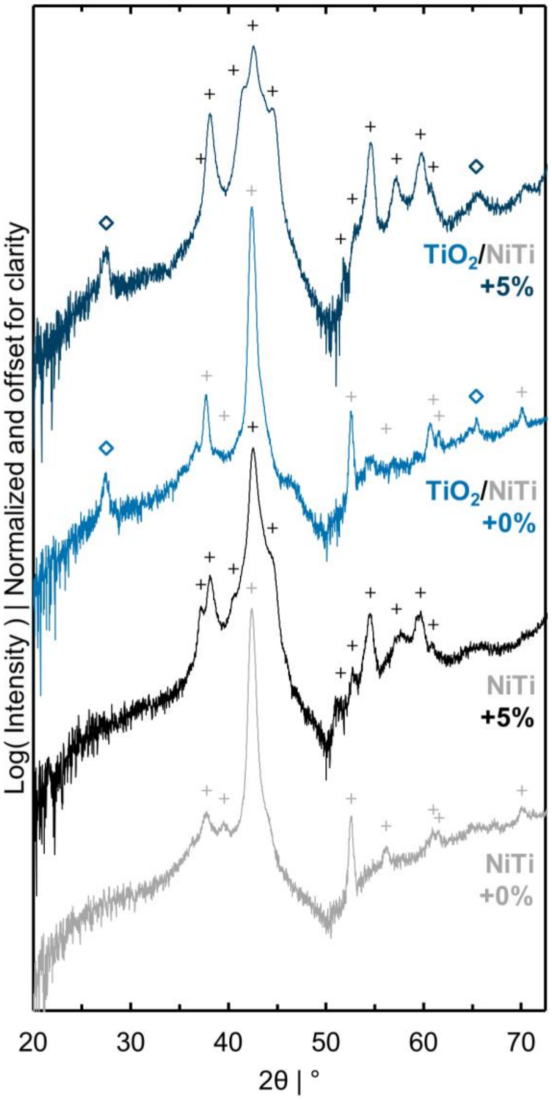

Figure S4. Survey XRD of NiTi with 0% (grey) and 5% (black) strain applied, and of TiO_2/NiTi with 0% (light blue) and 5% (dark blue) strain applied. Features corresponding to the bcc phase of NiTi (grey crosses) attenuate with increasing strain as new features corresponding to the monoclinic phase grow with increasing strain (black crosses). Beside this complex array of changing NiTi features, two distinct features correspond to the (110) and (310) planes of rutile TiO_2 appear in TiO_2/NiTi samples (denoted with dark blue diamonds).

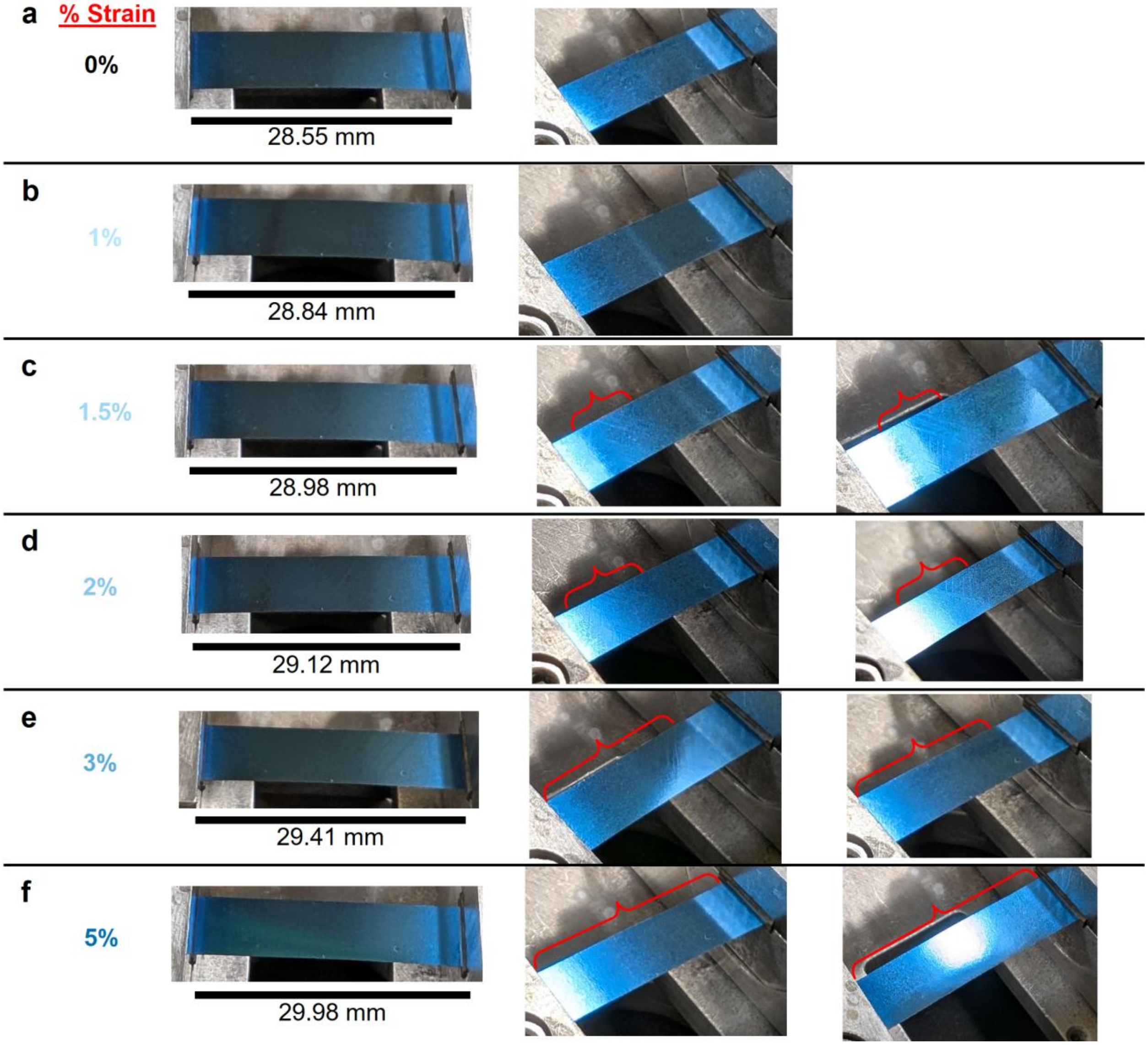

Figure S5. Images from several perspectives of TiO_2/NiTi under application of increasing strain for 0 (a), 1 (b), 1.5 (c), 2 (d), 3 (e), and 5% strain (f), where the red curly bracket denotes the region of the sample in which linear dislocations are visually observed. Linear dislocations increase in periodicity and decrease in parallelity as strain is increased above ~2%, leading to a cloudier (less reflective) appearance (compare the regions directly under the brackets for 1.5 and 2% strain for an example of this).

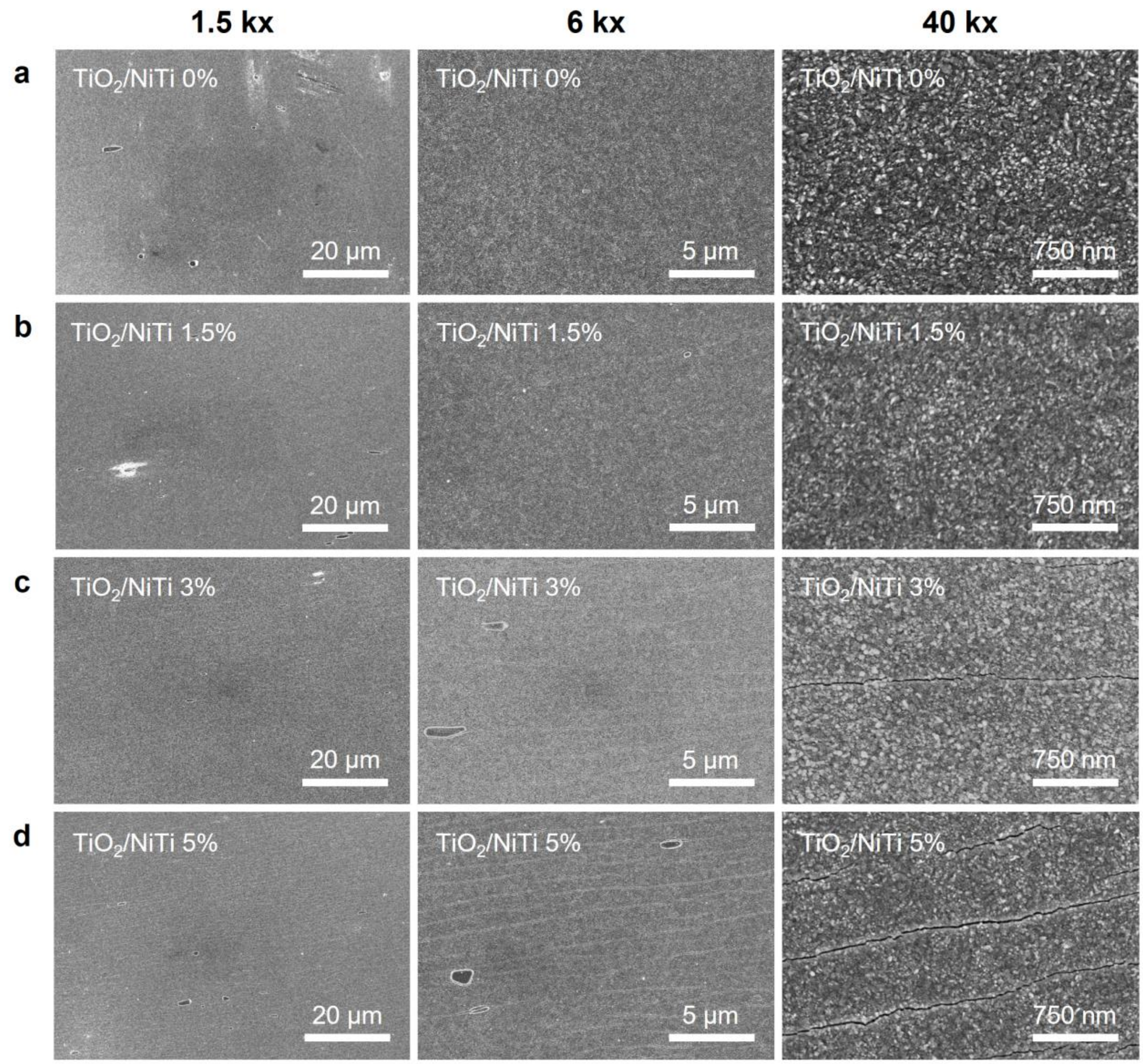

Figure S6. Normal-incidence scanning electron microscopy (SEM) images of TiO_2/NiTi strained to (a) 0%, (b) 1.5%, (c) 3%, and (d) 5% collected in a magnification series at 1.5 kx (left), 6 kx (center) and 50 kx (right). Cracks become increasingly apparent in the TiO_2 overlayer as increasing amounts of strain are applied.

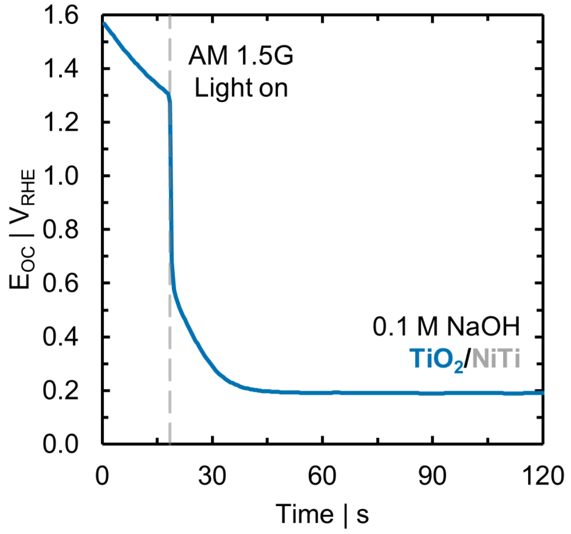

Figure S7. Open circuit potential for 0% strained TiO_2/NiTi in 0.1 M NaOH before (<18.5 s) and during AM 1.5G illumination (>18.5 s), where the cathodic shift in E_{oc} indicates n-type semiconducting properties. Vertical dashed grey line denotes the time at which AM 1.5G light was turned on.

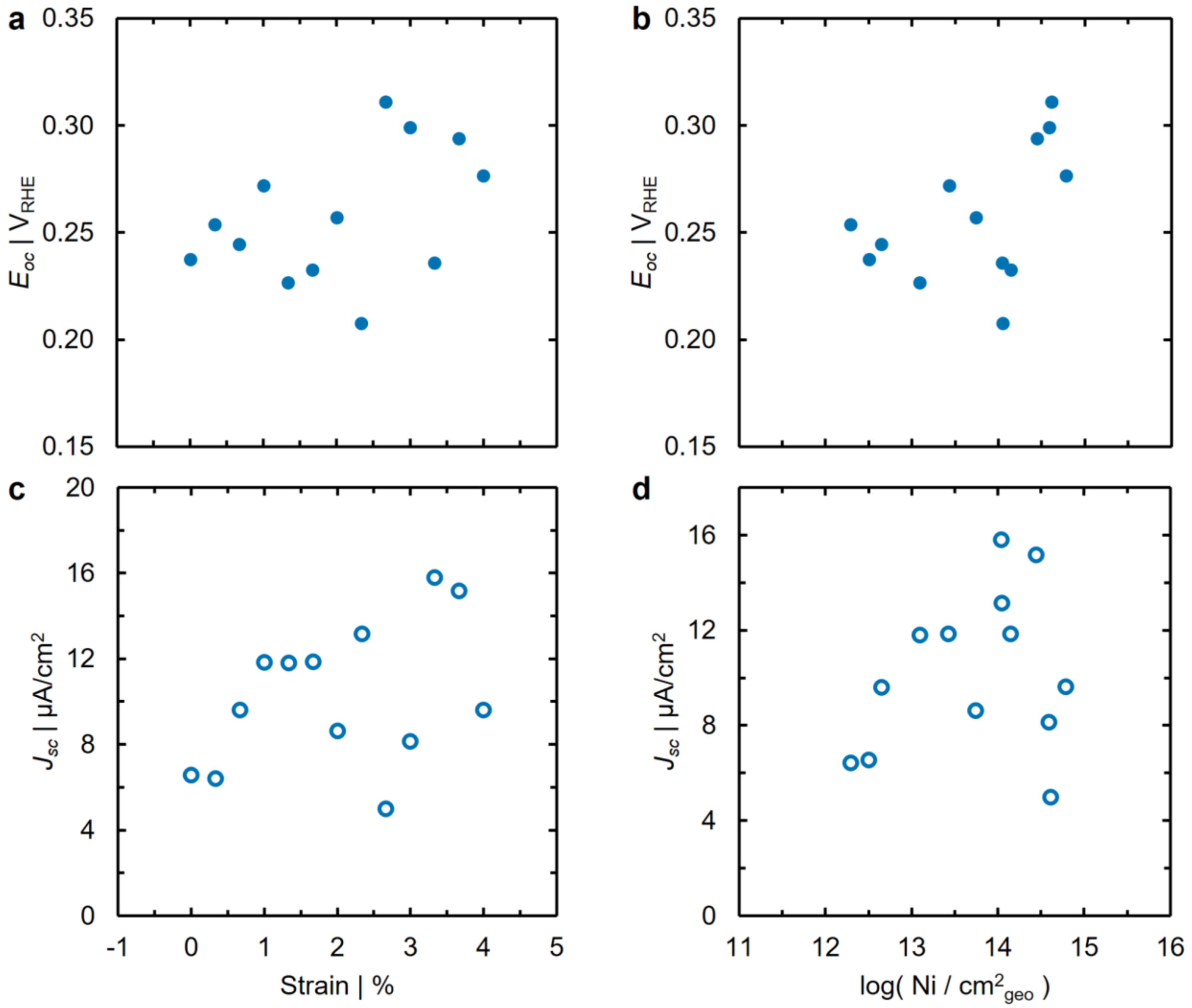

Figure S8. (a,b) Open circuit potential and (c,d) short circuit current density for TiO_2/NiTi as a function of (a,c) strain or (b,d) Ni site density on a logarithmic scale.

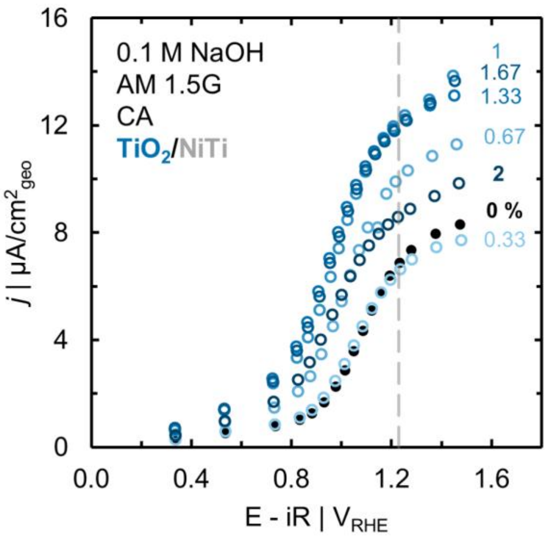

Figure S9. Steady-state current density as a function of potential obtained chronoamperometrically under AM 1.5 G illumination in 0.1 M NaOH for TiO_2/NiTi both unstrained (0%, solid black circle) and under increasing strain (blue open circles with increasingly dark shades denoting increasing strain).

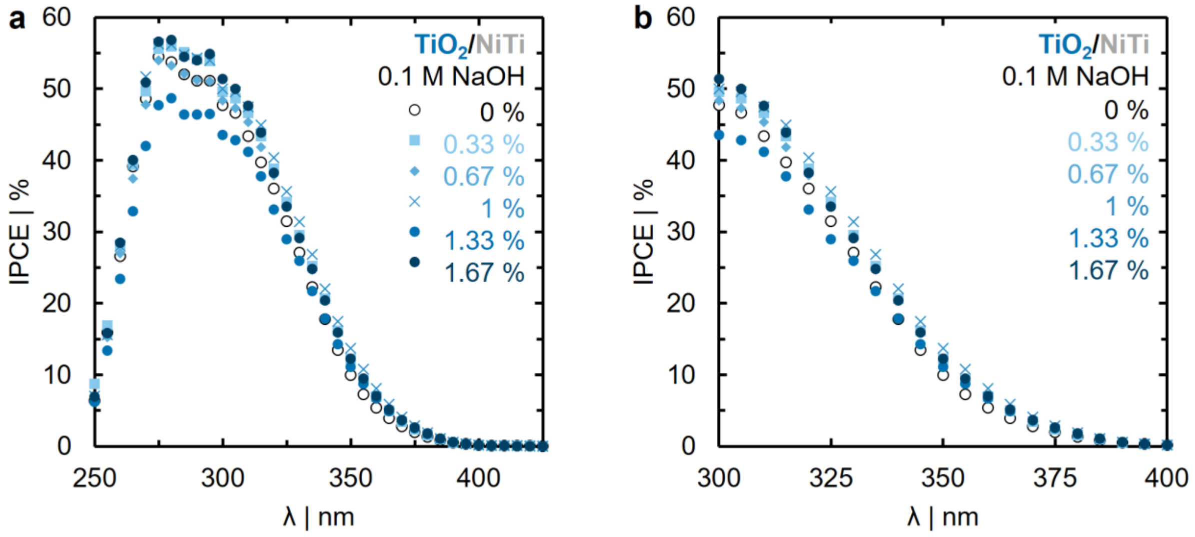

Figure S10. Incident-photon to current efficiency (IPCE), measured chronoamperometrically at 1.23 V_{RHE}, for unstrained (0%, open black circles) and strained TiO_2/NiTi (various symbols with denoted shades of blue). (a) Full spectrum and (b) zoom in to the absorption onset edge.

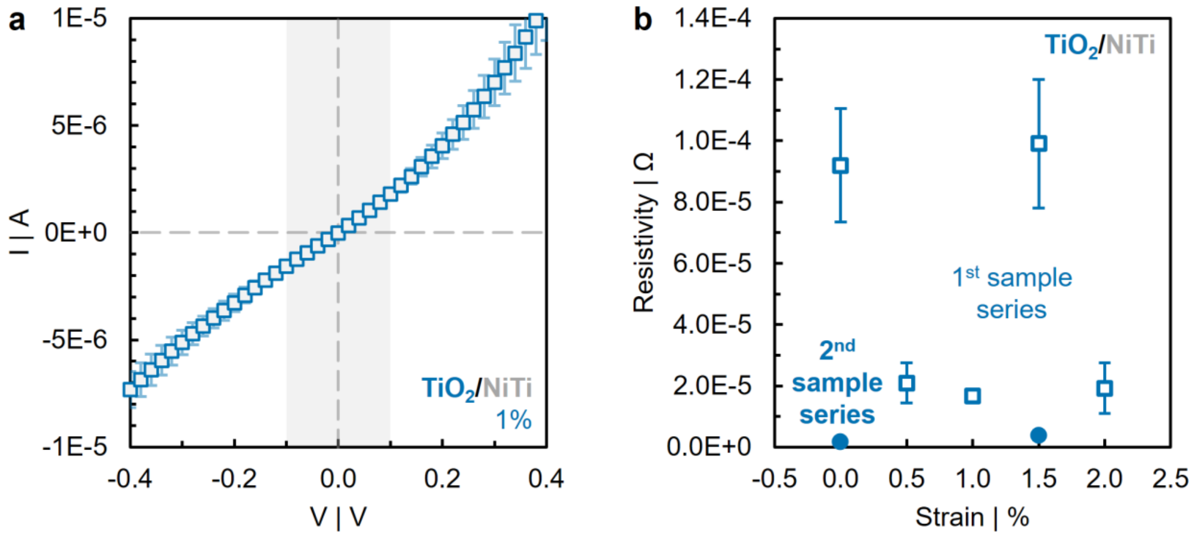

Figure S11. (a) Current-voltage response from a 2-point probe measurement of 1% strained TiO_2/NiTi, where data are reported as the average forward and reverse sweep currents measured at fixed potential for 8 cycles. Error bars denote plus and minus one standard deviation of the forward and reverse cycle average at each potential for all 8 cycles. The vertical grey bar denotes the region in which resistivity measurements were performed. (b) Resistivity as a function of mechanically applied strain for TiO_2/NiTi, denoted as the average (symbol) plus and minus the standard deviation of at least 8 cycles (error bars). Squares denote a first sample set, while the circles denote a second sample set testing the variability of samples for the 0 and 1.5% strained first samples.

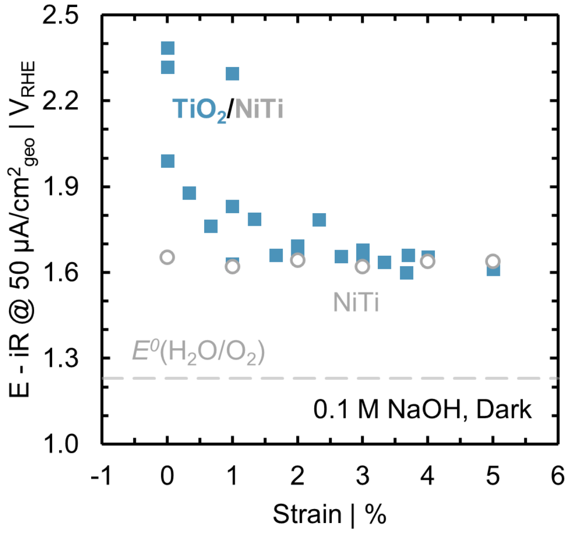

Figure S12. Electrochemical potential required to achieve 50 μA/cm$^2_{geo}$, extracted from cyclic voltammetry at 10 mV/s in 0.1 M NaOH in the dark, as a function of strain for TiO_2/NiTi (closed blue squares) and NiTi (open grey circles). Equilibrium potential for water oxidation denoted by horizontal grey dashed line.

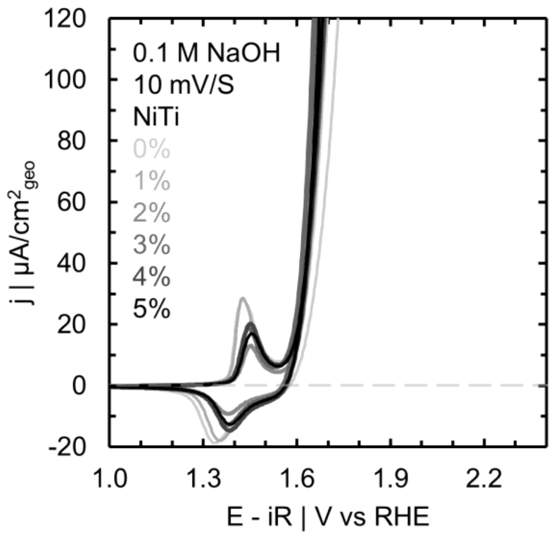

Figure S13. Cyclic voltammetry measured at 10 mV/s in dark 0.1 M NaOH of NiTi strained between 0 and 5% as denoted by increasingly dark shades of grey.

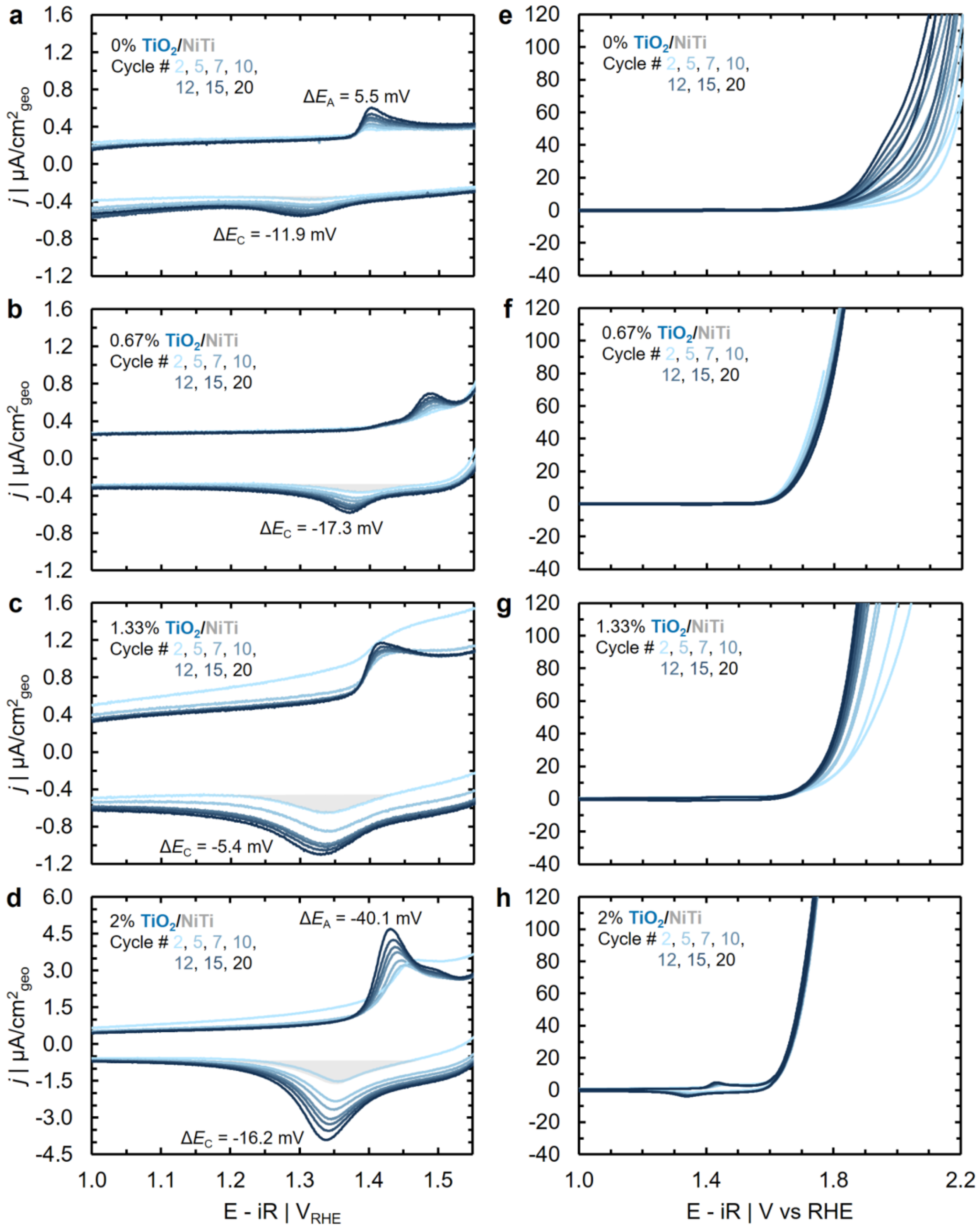

Figure S14. Cyclic voltammetry as a function of cycle number (denoted by increasingly dark shades of blue) of the double layer Ni^{3+}/Ni^{2+} redox features (a-d) and OER onset (e-h), measured at 50 mV/s in 0.1 M NaOH in the dark, for TiO_2/NiTi strained at 0% (a,e), 0.67% (b,f), 1.33% (c,g), and 2% strain (d,h). (a-d) Grey shade in cathodic-going Ni^{3+}/Ni^{2+} redox features provides an illustration of how redox features were integrated for measurement of Ni site density (actual integration measurements performed on the 3rd sweep of 10 mV/s data that follows the 50 mV/s cycling data shown here).

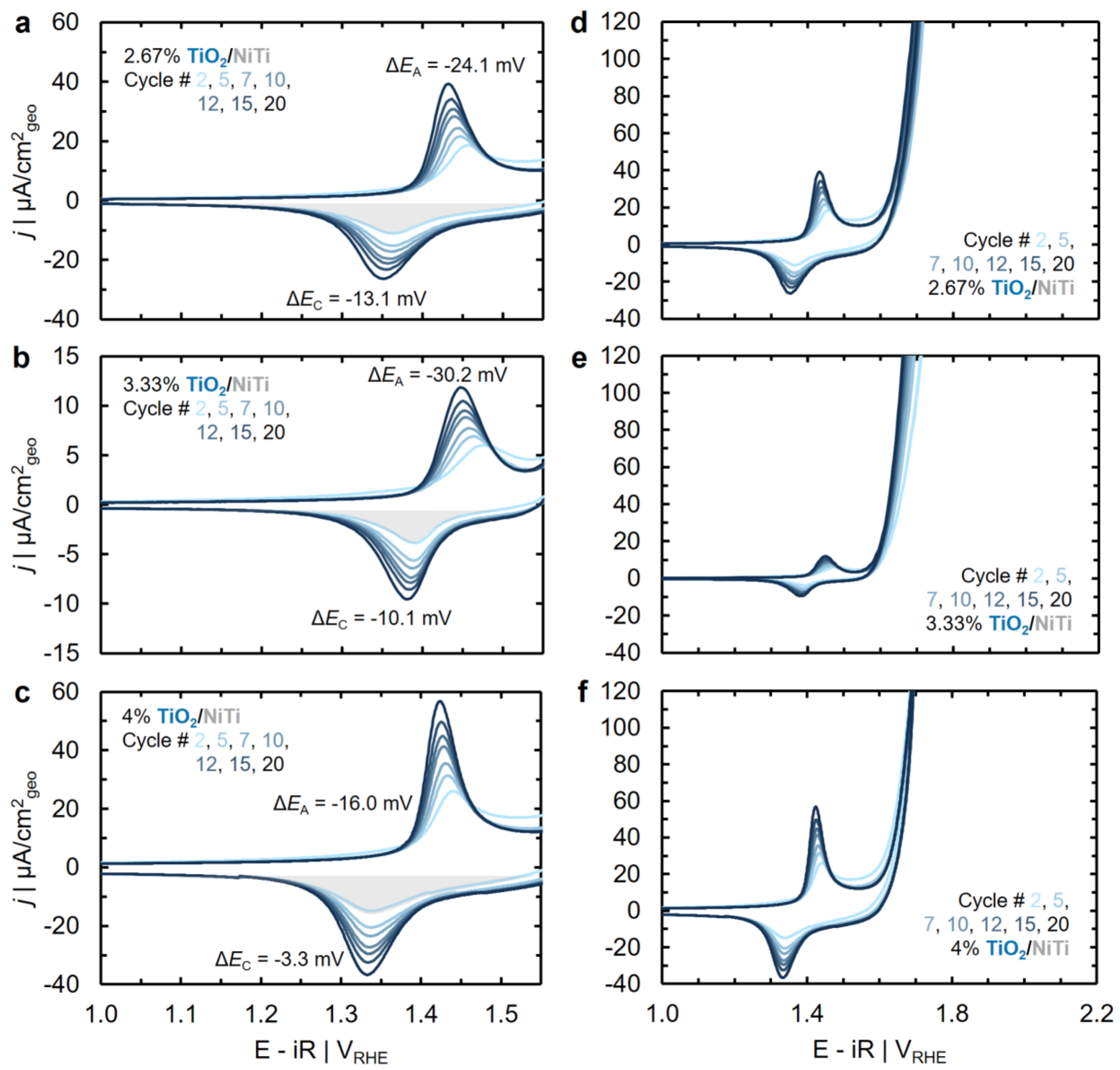

Figure S15. Cyclic voltammetry as a function of cycle number (denoted by increasingly dark shades of blue) of the double layer Ni^{3+}/Ni^{2+} redox features (a-d) and OER onset (d-f), measured at 50 mV/s in 0.1 M NaOH in the dark, for TiO_2/NiTi strained at 2.67% (a,d), 3.33% (b,e), and 4% strain (c,f). (a-c) Grey shade in cathodic-going Ni^{3+}/Ni^{2+} redox features provides an illustration of how redox features were integrated for measurement of Ni site density (actual integration measurements performed on the 3rd sweep of 10 mV/s data that follows the 50 mV/s cycling data shown here).

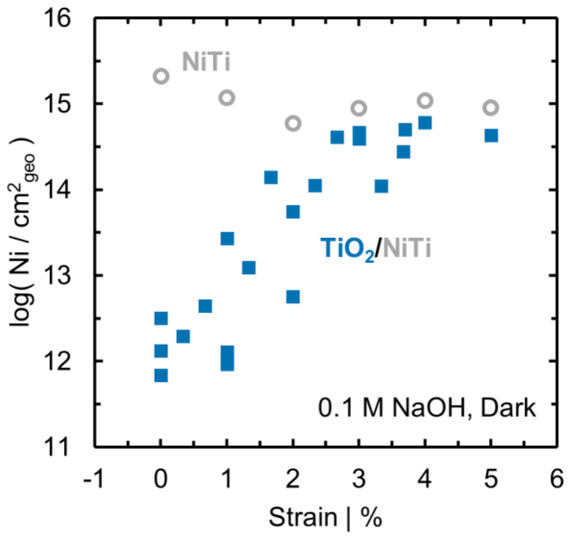

Figure S16. Ni site density on a logarithmic scale (log(Ni / cm^2_{geo})) versus strain for TiO_2/NiTi (closed blue squares) and NiTi (open grey circles), illustrating how Ni site density on TiO_2/NiTi asymptotically approaches that of the NiTi substrate as mechanically-applied strain increases.

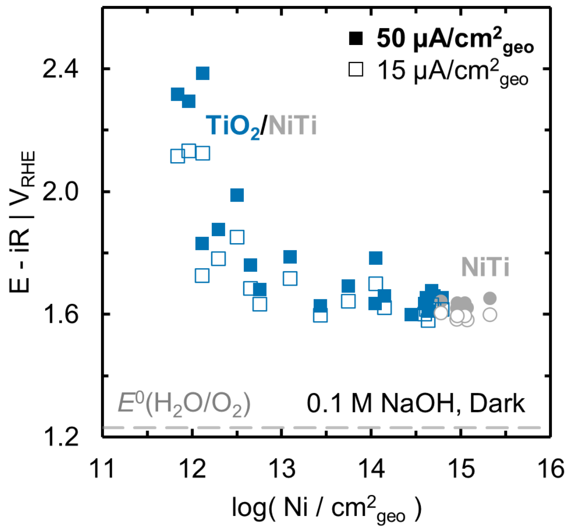

Figure S17. Dark OER overpotentials measured in 0.1 M NaOH at 50 (solid symbols) and 15 μA/cm^2_{geo} (open symbols) for TiO_2/NiTi (grey squares) and NiTi (blue circles). Trends between overpotential and Ni site density are preserved when varying the current defining overpotential within the kinetically-limited regime.

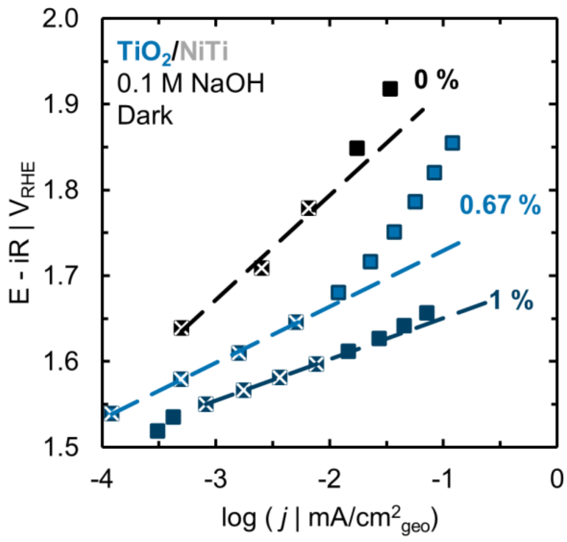

Figure S18. Examples of Tafel plots and slope analysis of chronoamperometric current density observed in 0.1 M NaOH in the dark on TiO_2/NiTi as a function of denoted strain. Squares with white x's denote data used to measure Tafel kinetics in a kinetically-limited regime.

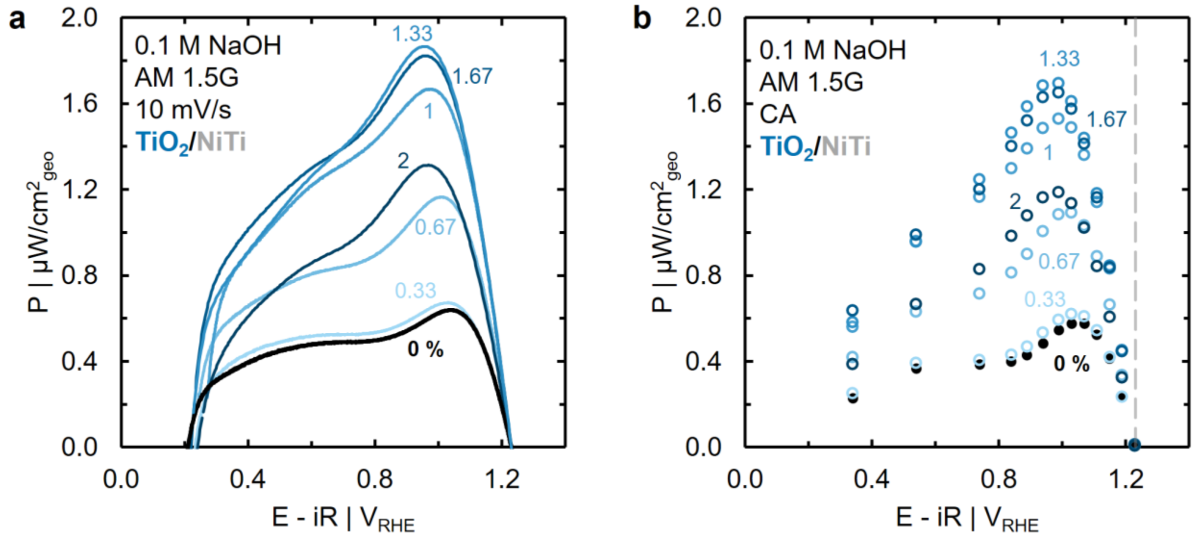

Figure S19. Power vs potential curves obtained by (a) cyclic voltammetry (CV) and chronoamperometrically (CA) from Figure S9 for TiO_2/NiTi both unstrained (0%, black (a) lines or (b) solid circles) and under increasing strain ((a) blue lines or (b) open circles, with increasingly dark shades denoting increasing strain) and TiO_2/Ti (grey (a) lines or (b) open squares).

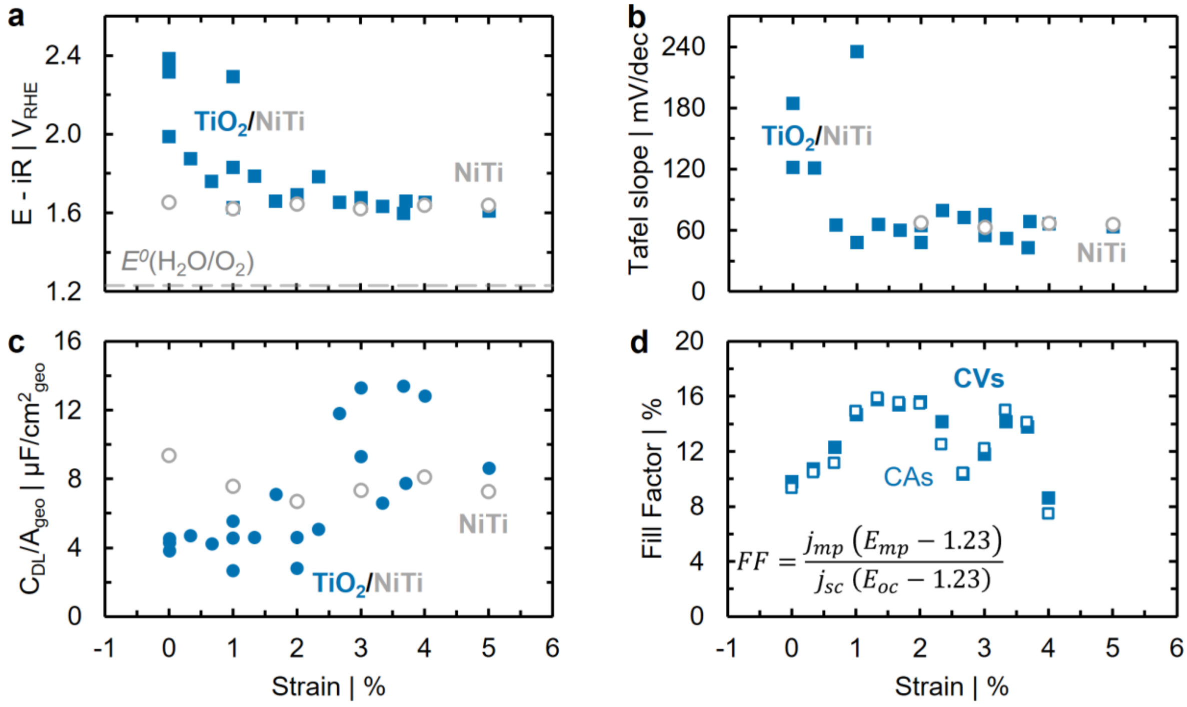

Figure S20. Role of strain on (a) overpotential required to generate 50 µA/cm$^2_{geo}$ of OER current, (b) Tafel slope, (c) double layer capacitance for TiO_2/NiTi (closed blue squares) and NiTi (open grey circles). (a) Horizontal grey dashed line denotes equilibrium redox potential for water oxidation. (d) Fill factor vs strain for TiO_2/NiTi collected by cyclic voltammetry (closed blue squares) or chronoamperometry (open blue circles). Instances where CV data appear missing are due to overlap of CV and CA data.

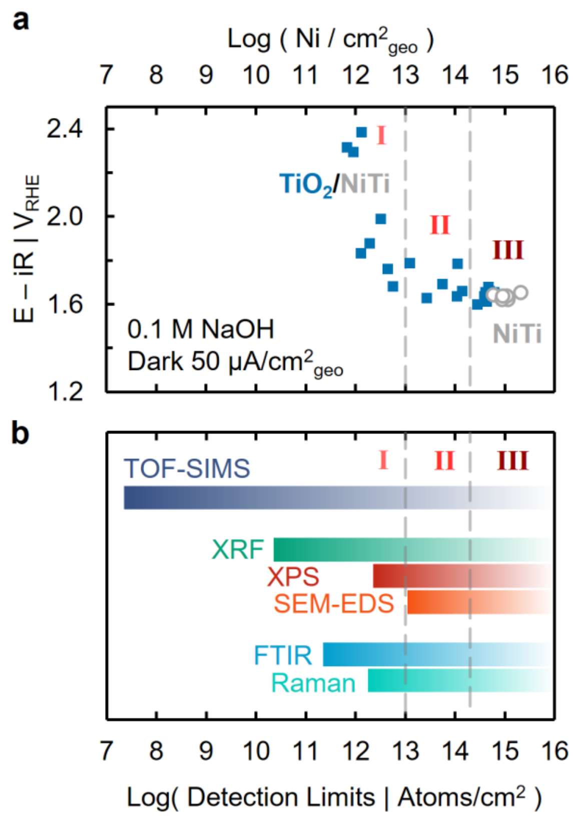

Figure S21. Comparison of (a) potential required to generate 50 µA/cm^2_{geo} in dark 0.1 M NaOH vs Ni site density on a log scale and (b) log-scale detection limits for several denoted techniques.[13]

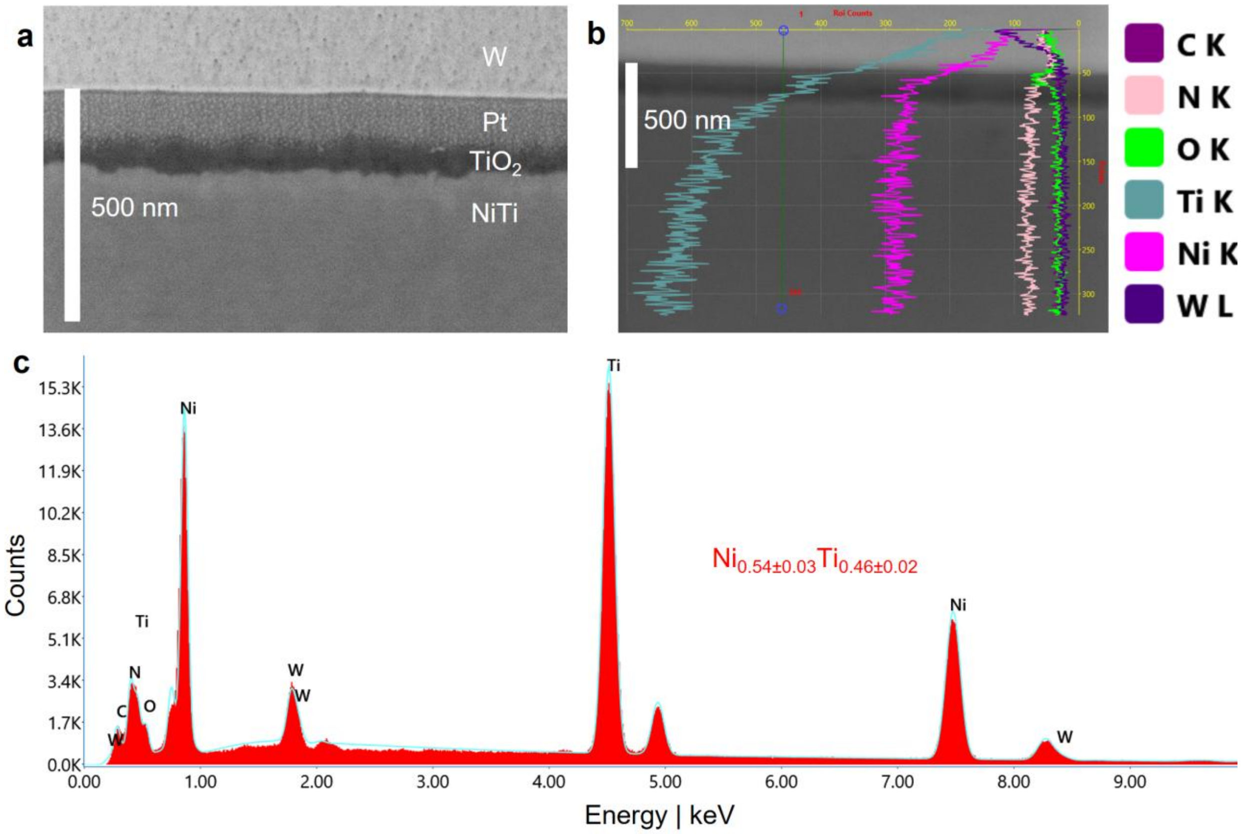

Figure S22. (a) Cross-sectional SEM image of TiO_2/NiTi collected with 2 kV electrons at 200,000x magnification with text denoting each separate layer of the TiO_2/NiTi heterostructure and Pt and W capping layers used to protect surface structure during focused ion beam (FIB) milling. (b) SEM-EDS line scan collected with 15 kV electrons at 150,000x magnification of the same cross section as in (a), illustrating Ti and Ni compositional heterogeneity throughout the near-surface and bulk of the TiO_2/NiTi heterostructure. Note the spatial resolution of SEM-EDS is on the order of several hundred nm under the high probe energies considered here, and therefore lacks the spatial resolution necessary to capture the discrete compositional changes occuring at the Pt/W, W/TiO_2 and TiO_2/NiTi interfaces observed with TOF-SIMS and STEM-EDS. (c) Detected counts vs energy corresponding to the line scan shown in (b), where an approximate composition of $Ni_{0.54\pm0.03}Ti_{0.46\pm0.02}$ is determined throughout the TiO_2/NiTi heterostructure and well into the NiTi bulk.

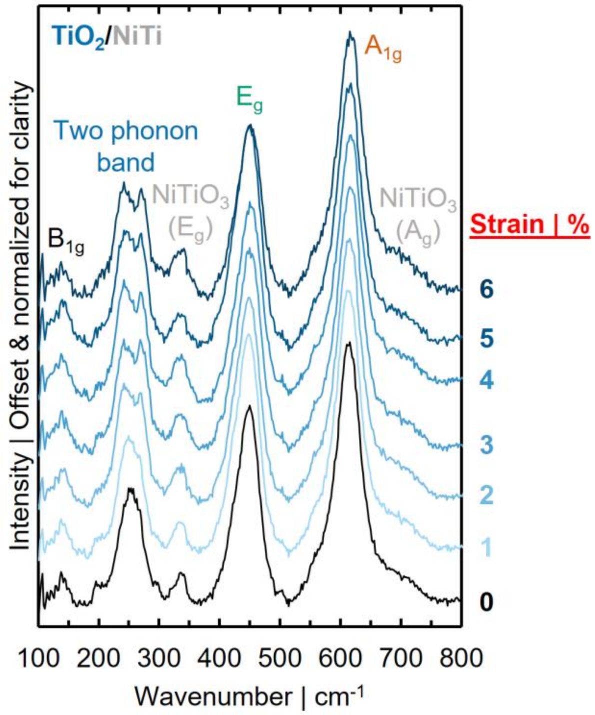

Figure S23. Raman spectra, collected with 532 nm incident light, of TiO_2/NiTi as a function of applied strain (denoted along right side of figure). Features denoted for rutile[14–16] TiO_2 in black (B_{1g}), blue (two-phonon mode), green (E_g), and orange (A_{1g}) and possible $NiTiO_3$ in grey[17,18] (E_g, A_g).

Supporting XPS Figures and SESSA Analysis

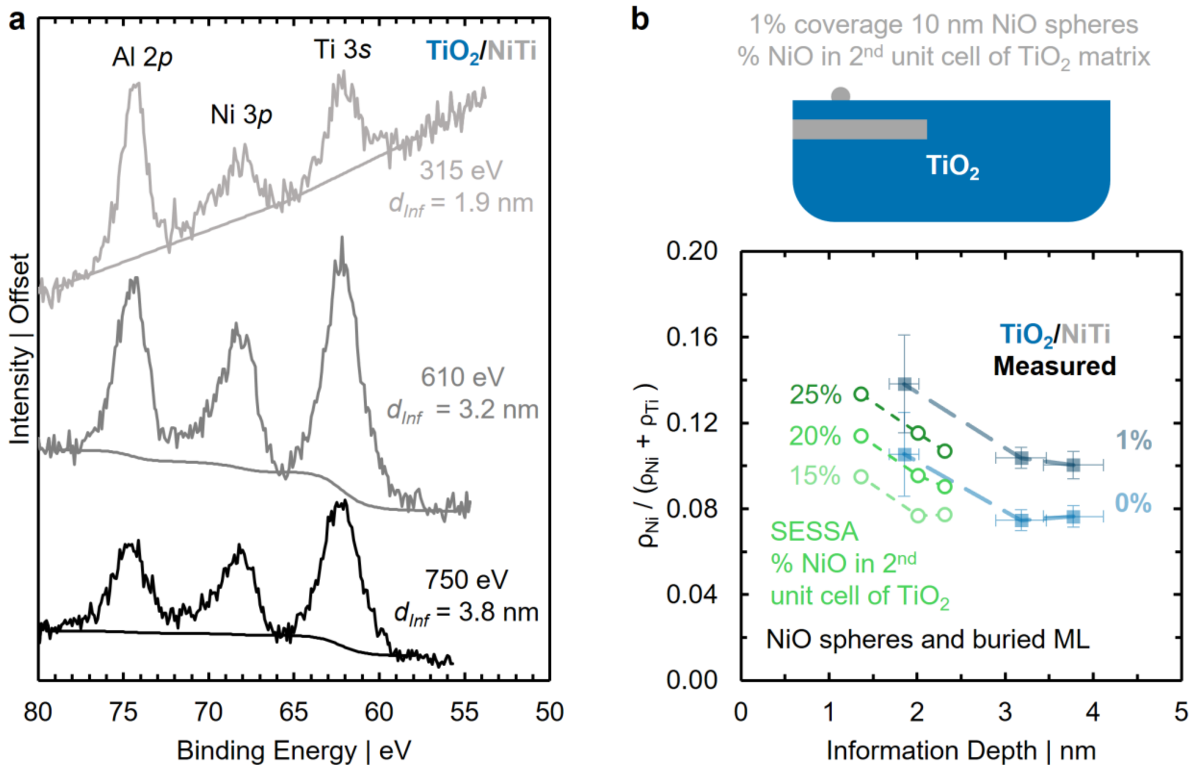

Figure S24. (a) Representative Al 2*p*, Ni 3*p*, and Ti 3*s* XPS core levels of 1% strained TiO_2/NiTi as a function of incident photon energies (and correspondingly information depths, d_{Inf}) at 750 eV (3.8 nm), 610 eV (3.2 nm), and 315 eV (1.9 nm). The Al 2*p* signal likely originates from residual polishing powder not removed by prior cleaning steps. Two Shirley backgrounds have been applied to data collected with 750 and 610 eV incident photon energy (one for Ti 3*s*, the other for Ni3*p* and Al 2*p*), while a pair of linear backgrounds have been applied to data collected at 315 eV, which ride along the low kinetic energy shoulder of a C *KLL* Auger feature. (b) Comparison of measured (filled blue squares and long dashed lines) and SESSA modelled (open green circles and short dashed lines) atomic density ratio of Ni to total metals (ρ_{Ni} / (ρ_{Ni} + ρ_{Ti})) based on Ni 3*p* and Ti 3*s* intensities for TiO_2/NiTi as a function of information depth.[19] Measured data are collected for 0% (light blue) and 1% strain (dark blue). Modelled SESSA data assume 1% coverage of 10 nm diameter NiO spheres at the surface and a series of denoted NiO compositions within the 2nd unit cell of a TiO_2 lattice. Error bars correspond to plus and minus one standard deviation for an effective 9% standard deviation for attenuation lengths ($\lambda_{AL} = 3d_{Inf}$) calculated using the CS-2 equation (eq. S3),[20] and the standard error for the ratio of standard deviations in XPS feature intensity derived from Monte Carlo analysis performed by CasaXPS.[21]

The ratio of Ni to total metal cation atomic densities (ρ_{Ni} / (ρ_{Ni} + ρ_{Ti})) is calculated assuming a semi-infinite specimen (*i*) while neglecting any attenuation effects from the overlayer, where the comparable kinetic energies of Ni 3*p* and Ti 3*s* core levels would be similarly attenuated by, e.g. a carbonaceous overlayer. Feature intensity ($N_i(\theta)$) is then defined as,[19]

$$N_i(\theta) = I_0(h\nu)T_0(E_{KE,i})\rho_i \frac{d\sigma_i}{d\Omega}(h\nu)\,\lambda_{AL,i}(E_{KE,i}) \tag{S1}$$

where $I_0(h\nu)$ is the X-ray flux as a function of incident photon energy ($h\nu$), $T_0(E_{KE})$ is the photoelectron kinetic energy (E_{KE}) dependent instrument transmission function, ρ_i is the atomic density [atoms/cm3] of the specimen, $d\sigma_i/d\Omega$ ($h\nu$) is the differential core level cross section, and $\lambda_{AL,i}(E_{KE})$ is the effective attenuation length of a photoelectron within the specimen. Taking the ratio of Eq. S1 for $N_{Ni\ 3p}$ to $N_{Ti\ 3s}$ measured at the same incident photon energy (so that the $I_0(h\nu)$ terms cancel), allows for the ratio of ρ_{Ni}/ρ_{Ti} to be solved for, where the $T_0(E_{KE})$ terms cancel due to the similar E_{KE} of Ni 3p and Ti 3s core levels and the remaining terms are defined as follows.

The differential core level cross section is calculated as,[19]

$$\frac{d\sigma_i}{d\Omega}(h\nu) = \frac{\sigma_i(h\nu)}{4\pi}\left[1 - \frac{\beta_i(h\nu)}{2}\left(\frac{3}{2}\sin^2(\alpha) - 1\right)\right] \tag{S2}$$

where σ_i and β_i are the $h\nu$ dependent core level cross section and asymmetry parameter,[22,23] and α is the angle between incident photon propagation and detected electron emission angle (75° for beamline 9.3.2).[24] Similarly, $\lambda_{AL,i}(E_{KE})$ is calculated in nm from the CS-2 equation,[20]

$$\lambda_{AL,i}(E_{KE}) = 0.316a^{\frac{3}{2}}\left\{\frac{E_{KE}}{Z^{0.45}\left[\ln\left(\frac{E_{KE}}{27}\right)+3\right]} + 4\right\} \tag{S3}$$

where Z is the average atomic number of the specimen and a is the calculated lattice parameter of the specimen in nm ($a = 10^7\left(MW_i\ /\ (D_i N_A)\right)^{\frac{1}{3}}$), where MW_i is the molecular weight of the specimen (g/mol), D_i is the density of the specimen (g/cm^3), and N_A is Avogadro's number.

The simple model used above to convert measured core level intensities into metal cation ratios assumes an atomically smooth and compositionally homogeneous surface, neither of which are true for the TiO_2/NiTi system considered here. While this simplified geometry lends itself to

modelling near-surface composition, it is unable to distinguish whether detected Ni signal originates from the true electrochemically active surface vs the inactive near-surface.

The National Institute for Standards and Technology (NIST), however, has developed the Simulation of Electron Spectra for Surface Analysis (SESSA) software for modelling XPS spectra of more complex surfaces.[25,26] When paired with the information depth tunability of synchrotron based XPS, SESSA allows users to model core level spectra based on hypothetical geometries and to compare them against measured spectral trends (Figure S24). For instance, by generating a surface containing a 1% surface coverage of 10 nm NiO spheres and variable Ni composition within the 2nd unit cell of TiO_2 (Figure S24b, top illustration), an information depth-dependent Ni compositional profile similar to that observed experimentally can be established (Figure S24b, green text and open circles).

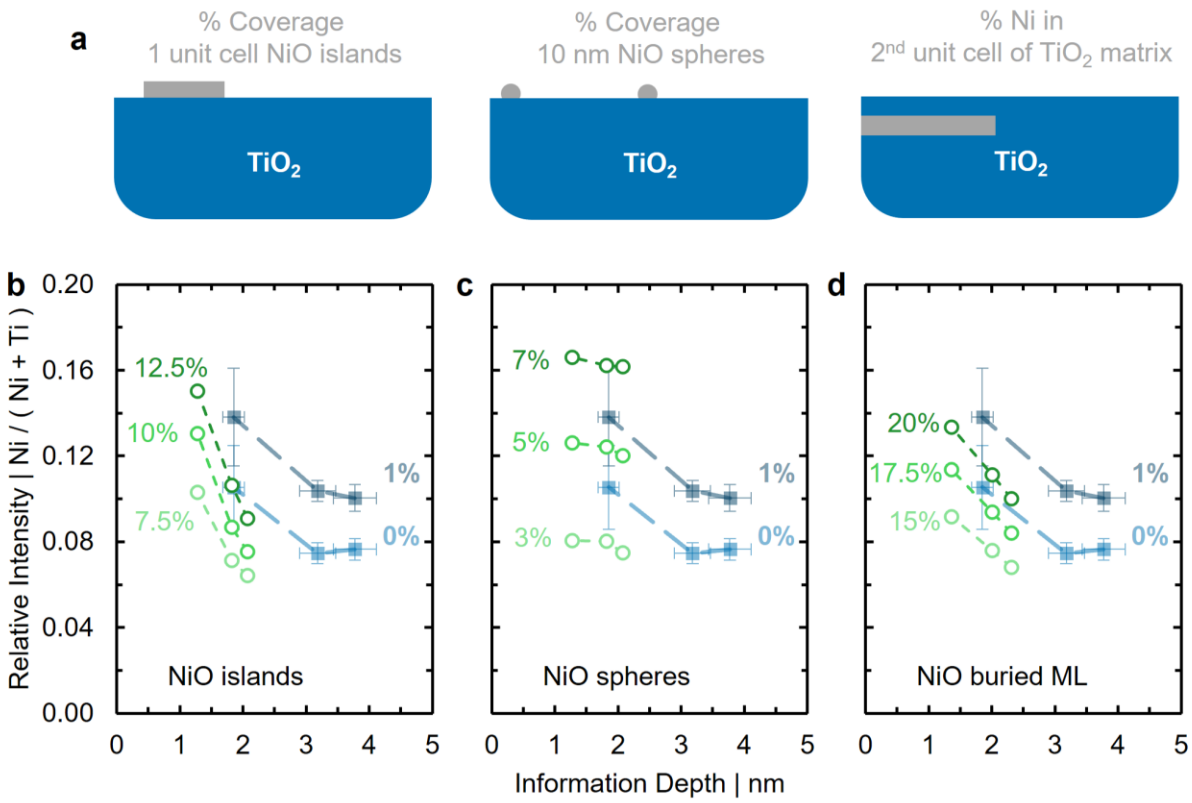

Figure S25. (a) Illustrations of the various models used to assess variable near surface composition of NiO (grey) and TiO_2 (blue). Experimental and modelled results of cross-section normalized metal cation ratios (Ni/(Ni + Ti)) can then be compared for (b) 1 unit cell thick rectangular islands of NiO with variable % coverage (denoted), (c) 10 nm NiO spheres recessed 2.5 nm into the TiO_2 surface at denoted coverages, and (d) assuming a denoted Ni composition buried within the 2nd unit cell of the TiO_2 matrix.

The complex mixture of NiO spheres and buried Ni composition within the 2nd unit cell of TiO_2 described in Figure S24 begins with defining a series of simplified geometries and comparing their depth-dependent compositional profiles against those determined experimentally (Figure S25). For instance, Ni composition attenuates too rapidly with depth when comparing 1 unit cell thick rectangular NiO islands to measured results (Figure S25b). In contrast, the modelled Ni composition of 10 nm NiO spheres varies too weakly against information depth to align with measured results (Figure S25c). Assuming Ni composition persists within the 2nd unit cell of TiO_2 provides a similar variation in Ni composition with probe depth as that observed experimentally, though lacks the plateau in Ni composition at deeper probing depths (Figure S25d). By combining

1% coverage of 10 nm NiO spheres with Ni composition buried within the 2nd unit cell of TiO_2, an information depth-dependent profile in Ni composition similar to that observed empirically is obtained, suggesting this more complex geometry is a more accurate representation of observed experimental results.

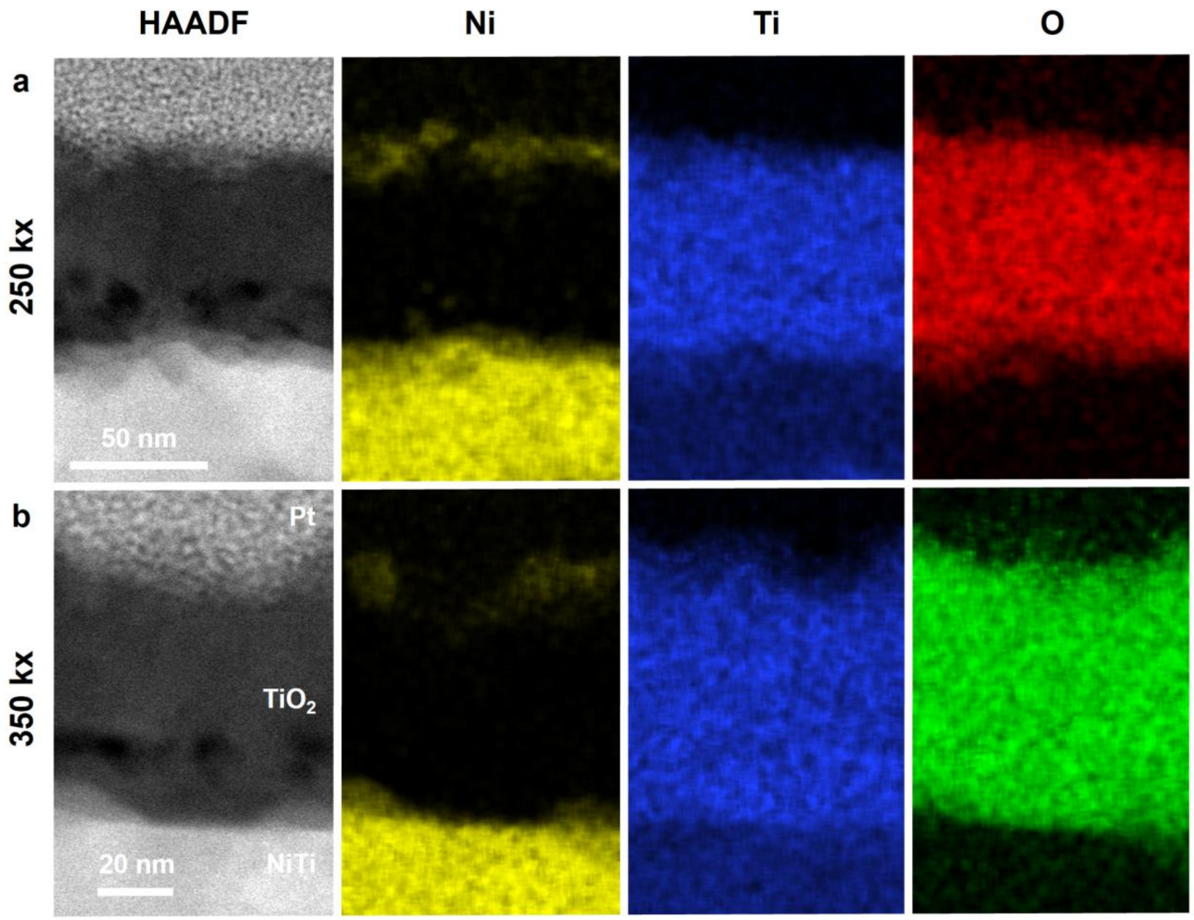

Figure S26. STEM HAADF and EDS maps of Ni, Ti, and O for TiO_2/NiTi at (a) 250 kx and (b) 350 kx magnification.

Supporting Information References

(1) Halldin Stenlid, J.; Görlin, M.; Diaz-Morales, O.; Davies, B.; Grigorev, V.; Degerman, D.; Kalinko, A.; Börner, M.; Shipilin, M.; Bauer, M.; et al. Operando Characterization of Fe in Doped $Ni_x(Fe_{1-x})O_yH_z$ Catalysts for Electrochemical Oxygen Evolution. *J. Am. Chem. Soc.* **2025**, *147*, 4120–4134.

(2) Trotochaud, L.; Ranney, J. K.; Williams, K. N.; Boettcher, S. W. Solution-Cast Metal Oxide Thin Film Electrocatalysts for Oxygen Evolution. *J. Am. Chem. Soc.* **2012**, *134*, 17253–17261.

(3) Lu, Z.; Xu, W.; Zhu, W.; Yang, Q.; Lei, X.; Liu, J.; Li, Y.; Sun, X.; Duan, X. Three-Dimensional NiFe Layered Double Hydroxide Film for High-Efficiency Oxygen Evolution Reaction. *Chem. Commun.* **2014**, *50*, 6479–6482.

(4) Louie, M. W.; Bell, A. T. An Investigation of Thin-Film Ni–Fe Oxide Catalysts for the Electrochemical Evolution of Oxygen. *J. Am. Chem. Soc.* **2013**, *135*, 12329–12337.

(5) Klaus, S.; Cai, Y.; Louie, M. W.; Trotochaud, L.; Bell, A. T. Effects of Fe Electrolyte Impurities on $Ni(OH)_2$/NiOOH Structure and Oxygen Evolution Activity. *J. Phys. Chem. C* **2015**, *119*, 7243–7254.

(6) Diaz-Morales, O.; Ferrus-Suspedra, D.; M. Koper, M. T. The Importance of Nickel Oxyhydroxide Deprotonation on Its Activity towards Electrochemical Water Oxidation. *Chemical Science* **2016**, *7*, 2639–2645.

(7) Corrigan, D. A. The Catalysis of the Oxygen Evolution Reaction by Iron Impurities in Thin Film Nickel Oxide Electrodes. *Journal of The Electrochemical Society* **1987**, *134*, 377.

(8) Trotochaud, L.; Young, S. L.; Ranney, J. K.; Boettcher, S. W. Nickel–Iron Oxyhydroxide Oxygen-Evolution Electrocatalysts: The Role of Intentional and Incidental Iron Incorporation. *J. Am. Chem. Soc.* **2014**, *136*, 6744–6753.

(9) Radinger, H.; Connor, P.; Tengeler, S.; Stark, R. W.; Jaegermann, W.; Kaiser, B. Importance of Nickel Oxide Lattice Defects for Efficient Oxygen Evolution Reaction. *Chem. Mater.* **2021**, *33*, 8259–8266.

(10) Alsabet, M.; Grden, M.; Jerkiewicz, G. Electrochemical Growth of Surface Oxides on Nickel. Part 1: Formation of α-$Ni(OH)_2$ in Relation to the Polarization Potential, Polarization Time, and Temperature. *Electrocatal* **2011**, *2*, 317–330.

(11) Hahn, F.; Floner, D.; Beden, B.; Lamy, C. *In Situ* Investigation of the Behaviour of a Nickel Electrode in Alkaline Solution by *Uv-Vis* and *Ir* Reflectance Spectroscopies. *Electrochimica Acta* **1987**, *32*, 1631–1636.

(12) Standard Tables for Reference Solar Spectral Irradiances: Direct Normal and Hemispherical on 37 Tilted Surface, 2020.

(13) Eswara, S.; Pshenova, A.; Yedra, L.; Hoang, Q. H.; Lovric, J.; Philipp, P.; Wirtz, T. Correlative Microscopy Combining Transmission Electron Microscopy and Secondary Ion Mass Spectrometry: A General Review on the State-of-the-Art, Recent Developments, and Prospects. *Applied Physics Reviews* **2019**, *6*, 021312.

(14) Porto, S. P. S.; Fleury, P. A.; Damen, T. C. Raman Spectra of TiO_2, MgF_2, ZnF_2, FeF_2, and MnF_2. *Phys. Rev.* **1967**, *154*, 522–526.

(15) Balachandran, U.; Eror, N. G. Raman Spectra of Titanium Dioxide. *Journal of Solid State Chemistry* **1982**, *42*, 276–282.

(16) Frank, O.; Zukalova, M.; Laskova, B.; Kürti, J.; Koltai, J.; Kavan, L. Raman Spectra of Titanium Dioxide (Anatase, Rutile) with Identified Oxygen Isotopes (16, 17, 18). *Phys. Chem. Chem. Phys.* **2012**, *14*, 14567–14572.
(17) Chuang, S.-H.; Hsieh, M.-L.; Wu, S.-C.; Lin, H.-C.; Chao, T.-S.; Hou, T.-H. Fabrication and Characterization of High-k Dielectric Nickel Titanate Thin Films Using a Modified Sol–Gel Method. *Journal of the American Ceramic Society* **2011**, *94*, 250–254.
(18) Bellam, J. B.; Ruiz-Preciado, M. A.; Edely, M.; Szade, J.; Jouanneaux, A.; Kassiba, A. H. Visible-Light Photocatalytic Activity of Nitrogen-Doped $NiTiO_3$ Thin Films Prepared by a Co-Sputtering Process. *RSC Adv.* **2015**, *5*, 10551–10559.
(19) Fadley, C. S. Basic Concepts of X-Ray Photoelectron Spectroscopy. In *Electron Spectroscopy: Theory, Techniques, and Applications*; Academic Press: New York, 1978; Vol. 2, pp 1–156.
(20) Cumpson, P. J.; Seah, M. P. Elastic Scattering Corrections in AES and XPS. II. Estimating Attenuation Lengths and Conditions Required for Their Valid Use in Overlayer/Substrate Experiments. *Surface and Interface Analysis* **1997**, *25*, 430–446.
(21) Fairley, N.; Fernandez, V.; Richard-Plouet, M.; Guillot-Deudon, C.; Walton, J.; Smith, E.; Flahaut, D.; Greiner, M.; Biesinger, M.; Tougaard, S.; et al. Systematic and Collaborative Approach to Problem Solving Using X-Ray Photoelectron Spectroscopy. *Applied Surface Science Advances* **2021**, *5*, 100112.
(22) Yeh, J. J.; Lindau, I. Atomic Subshell Photoionization Cross Sections and Asymmetry Parameters: $1 \leqslant Z \leqslant 103$. *Atomic Data and Nuclear Data Tables* **1985**, *32*, 1–155.
(23) Yeh, J.-J. *Atomic Calculation of Photoionization Cross-Sections and Asymmetry Parameters*; Gordon & Breach Science, Publishers, 1993.
(24) Grass, M. E.; Karlsson, P. G.; Aksoy, F.; Lundqvist, M.; Wannberg, B.; Mun, B. S.; Hussain, Z.; Liu, Z. New Ambient Pressure Photoemission Endstation at Advanced Light Source Beamline 9.3.2. *Review of Scientific Instruments* **2010**, *81*, 053106.
(25) Smekal, W.; Werner, W. S. M.; Powell, C. J. Simulation of Electron Spectra for Surface Analysis (SESSA): A Novel Software Tool for Quantitative Auger-Electron Spectroscopy and X-Ray Photoelectron Spectroscopy. *Surface and Interface Analysis* **2005**, *37*, 1059–1067.
(26) Powell, C. J. NIST Standard Reference Database 100: NIST Database for the Simulation of Electron Spectra for Surface Analysis (SESSA), 2024. https://www.nist.gov/srd/nist-standard-reference-database-100.
